# Therapeutic options for advanced epidermal growth factor receptor (EGFR)-mutant non-small cell lung cancer: a Bayesian network secondary analysis

**DOI:** 10.18632/aging.103066

**Published:** 2020-04-23

**Authors:** Xinmin Zeng, Xuan Wan, Jun Xu, Hui Wang, Hua Chen, Qinghua Zeng, Wenxiong Zhang, Binghao Zhao

**Affiliations:** 1 Department of Thoracic Surgery, Nanchang First Hospital, Nanchang 330008, China; 2 Department of Pulmonary and Critical Care Medicine, The First Affiliated Hospital of Nanchang University, Nanchang 330006, China; 3 Department of Oncology, Nanchang First Hospital, Nanchang 330008, China; 4 Department of Thoracic Surgery, The Second Affiliated Hospital of Nanchang University, Nanchang 330006, China; 5 Department of Neurosurgery, Peking Union Medical College Hospital, Chinese Academy of Medical Sciences and Peking Union Medical College, Beijing 100730, China

**Keywords:** advanced EGFR-mutant NSCLC, effective options, Bayesian study

## Abstract

The most favorable treatments for advanced EGFR-mutant NSCLC are less indicated. Forty-one studies were eligible for this Bayesian network secondary analysis. For PFS, erlotinib (Erlo)+bevacizumab (Bev) (HR 0.26, 95% CrI: 0.08-0.75 vs placebo), osimertinib (Osi) (HR 0.29, 0.11-0.70 vs placebo), and afatinib (Afa) were top-ranking individual treatments, while immunotherapy (IT)+anti-VEGFR (aVEGFR)+platinum-based therapy (Plat) (HR 0.42, 0.06-2.63 vs placebo), EGFR-TKI (ET)+aVEGFR (HR 0.35, 0.14-0.85 vs placebo), and ET+aVEGFR+Plat were top-ranking medication classes. For OS, Osi (HR 0.52, 0.10-2.00 vs placebo), cetuximab (Cet)+Bev+Plat (HR 0.51, 0.06-3.38 vs placebo), and cilengitide (Cil)+Cet+Plat were top-ranking individual treatments, while ET+aVEGFR+Plat, ET+Plat, and third-generation EGFR-TKI (3^rd^ ET) were top-ranking medication classes. For PFS regarding the EGFR genomic aberration status, Erlo+Bev, Osi, and Afa were superior for exon 19 deletion status, whereas ET+Bev, Osi, and gefitinib (Gef)+pemetrexed (Peme) were excellent for exon 21 L858Arg mutation status. The results were consistent in terms of the ORR and DoR and remained robust across sensitivity analyses. However, Erlo + Bev had the most grade 3 or higher adverse events. Osi, Erlo+Bev, and Erlo+Bev+Plat are reasonably recommended to balance PFS and OS, but adverse events should be considered. IT+aVEGFR+Plat shows potential superiority, but more clinical evidence is needed.

## INTRODUCTION

Non-small cell lung cancer (NSCLC) represents approximately 85% to 90% of lung cancer cases and is the leading cause of cancer-related death worldwide, with a lower than 15% 5-year survival [1, 2]. Since treatment selections have become increasingly related to the biological subtypes of NSCLC, attention has been drawn to tumors harboring epidermal growth factor receptor (EGFR) mutations, which are estimated to exist in 10%-15% of patients with nonsquamous NSCLC [3]. The identification of EGFR mutations has led to the development of targeted therapies, including small molecule tyrosine kinase inhibitors (TKIs) directed at the signal transduction pathway as well as immunotherapies incorporating checkpoint monoclonal antibodies that bind to and inactivate the receptors on cell membranes [4].

As a monotherapy, gefitinib, erlotinib and, more recently, afatinib have been licensed and recommended as first-line treatment regimens for EGFR-mutant NSCLC patients by the European Society for Medical Oncology (ESMO) guidelines. In August 2015, the American Society of Clinical Oncology (ASCO) clinical guidelines recommended two cytotoxic drugs, docetaxel and pemetrexed, and two EGFR-TKIs, erlotinib and gefitinib, to patients who experienced treatment failure with conventional first-line chemotherapy [4]. Nevertheless, several new regimens have been approved by the US FDA, such as the combination of docetaxel and ramucirumab, nivolumab, pembrolizumab, and atezolizumab. At the same time, more than 40 therapeutic options are being assessed in randomized controlled trials (RCTs) [5]. With more clinical trials emerging [4–46], the FLAURA trial [42] has shown that osimertinib has superior efficacy compared with standard EGFR-TKIs in treating advanced EGFR-mutant NSCLC with less serious adverse effects (18.9 months vs 10.2 months for progression-free survival (PFS), P<0.001). The newest National Comprehensive Cancer Network (NCCN) guidelines also regarded osimertinib as category 1 for advanced EGFR-mutant NSCLC.

There is an urgent need to identify complete information on the most effective and latest treatment for advanced EGFR-mutant NSCLC. Conventional meta-analyses have only partially captured the available evidence for treating the intended populations; their outcomes are not comprehensive. This work is a generalized version of a pairwise meta-analysis integrating direct and indirect evidence [4–46] to aid in clinical decision making. Thus, the aim of this article is to comprehensively evaluate the effectiveness and safety of various therapeutics for advanced EGFR-mutant NSCLC.

## RESULTS

### Study selection and characteristics of the included studies

We identified a total of 1749 records from a database search and 34 records from other available literature; of these, 1721 were excluded based on the selection criteria. Subsequently, 62 potential articles went through full-text review, and 41 studies were ultimately eligible for inclusion (Appendix Figure 1 in the Supplementary Data).

Forty-one RCTs [6–46] consisting of 8430 total participants were included in the analysis. The characteristics and results of the studies are detailed in Appendix Table 2 in the Supplementary Data. The included RCTs encompassed 22 unique treatments and 15 medication classes; there were 39 direct comparisons for PFS (38 trials [6–24, 26–28, 30–40, 42–46]; n= 7670) and 23 direct comparisons for OS (22 trials [6, 7, 9, 11–13, 15, 19–22, 25, 27, 29, 35, 36, 38–42, 45]; n= 3842). Data were extracted from survival plots in 5 studies [9, 11, 18, 22, 35]. Most treatments in eligible trials were first-line setting, the abbreviations for the medication classes and their constituent individual treatments are listed in Table 1. The mean age of the participants ranged from 56.0 to 74.0 years, with a median age of 61.9 years. The follow-up period ranged from 10 to 70 months with a median duration of 27.5 months. Ten studies [14, 26, 31, 34, 35, 38, 39, 42, 43, 46] reported data on metastases, 9 [14, 26, 34, 35, 38, 39, 42, 43, 46] reported data on central nervous system (CNS) metastases, and 5 [14, 26, 31, 34, 42] reported data on visceral, bone or other metastases. Of the 41 total studies, 40 were two-arm trials, 1 [45] was a three-arm trial, 34 studies were phase III clinical trials, and 7 [6, 7, 18, 22, 27, 30, 31] were phase II trials. Of note, 16 studies [10, 14, 22, 23, 28–32, 34, 35, 38, 39, 41, 42, 46] provided EGFR genomic aberration data (19 del and/or 21 L858R), 14 [10, 14, 22, 23, 28, 30–32, 34, 35, 38, 39, 42, 46] for PFS and 2 [29, 41] for OS. There were 12 trials involving Asian patients and 29 trials involving 6408 non-Asian patients (multiple nations or no Asian region).

**Table 1:** List of medication classes and individual treatments.

**Medication class level**	**Treatment level**
**1^st^-gen ET**: first generation EGFR-TKI	**Gef**: gefitinib
	**Erlo**: erlotinib
	**Ico**: icotinib
**2^nd^-gen ET**: second generation EGFR-TKI	**Afa**: afatinib
	**Dac**: dacomitinib
**3^rd^-gen ET**: third generation EGFR-TKI	**Osi**: osimertinib
	**Naq**: naquotinib
**ET+aVEGFR**: EGFR-TKI+anti-VEGFR	**Erlo+Bev**: erlotinib+bevacizumab
	**Sun+Erlo**: sunitinib+erlotinib
**MT+ET**: MET-TKI+EGFR-TKI	**Ona+Erlo**: onartuzumab+erlotinib
	**Erlo+Tiv**: erlotinib+tivantinib
**ET+CT**: EGFR-TKI+cytotoxic therapy	**Gef+Peme**: gefitinib+pemetrexed
**ET+aVEGFR+Plat**: EGFR-TKI+anti-VEGFR + platinum-based therapy	**Cil+Cet+Plat**: cilengitide+cetuximab+platinum-based therapy
	**Cet+Bev+Plat**: cetuximab+bevacizumab+platinum-based therapy
**ET+Plat**: EGFR-TKI+platinum-based therapy	**Cet+Plat**: cetuximab+platinum-based therapy
	**Erlo+Plat**: erlotinib+platinum-based therapy
**aVEGFR+Plat**: anti-VEGFR+platinum-based therapy	**Mot+Plat**: motesanib+platinum-based therapy
Plat*	**Plat**: platinum-based therapy
**CT**: cytotoxic therapy	**Doc**: docetaxel
	**Vin**: vinorelbine
**WBRT**	**WBRT**: whole-brain radiotherapy
**Placebo**	Placebo

*In this study, platinum-based therapy contains: pemetrexed + cisplatin/carboplatin, gemcitabine + cisplatin/carboplatin, vinorelbine + cisplatin/carboplatin, paclitaxel + cisplatin/carboplatin, docetaxel + cisplatin/carboplatin, docetaxel + gemcitabine + cisplatin/carboplatin, pemetrexed + gemcitabine+cisplatin/carboplatin.

### Risk of bias and quality assessment

In the quality assessment, we found that a vast majority of the included studies had a low risk of bias (Appendix Table 3 in the Supplementary Data). Two [24, 46] studies had “other bias” according to the Cochrane risk of bias tool, and 16 [10, 13, 15, 19, 20, 22, 26–29, 31, 35, 38, 39, 41, 44] had an unclear “other bias”. Most of the risk stemmed from the blinding of participants and personnel and blinding of outcome assessment categories due to the open-label method.

### Bayesian NMA at the individual-treatment level

**Figure 1:**
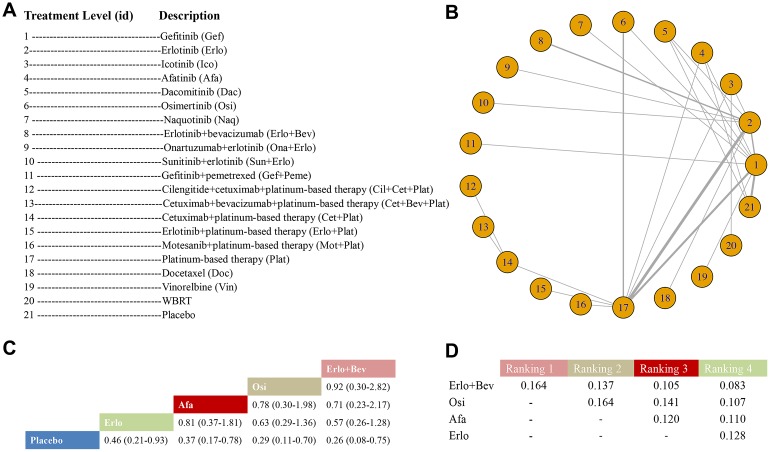
**Meta-analysis of efficacy for PFS at the individual-treatment level.** (**A**) Descriptions of the individual treatments included in this analysis. (**B**) Network plots showing comparisons between nodes (yellow circles), each representing a unique individual treatment. Each line corresponds to direct comparisons between treatments with the width corresponding to the number of direct within-trial comparisons. (**C**) Comparison results of the most efficacious treatments and placebo (HR (95% CrI)). Each result is a comparison between the column-defining drug and the row-defining treatment. (**D**) Schematic detailing the most efficacious treatments according to the rankograms.

A total of 37 RCTs with 37 arms and 21 unique treatment levels were included in the treatment level analysis for PFS; a study by Reck et al. [45] was omitted for not contributing to a complete loop (Figure 1A). Twenty-one unique nodes were presented in this analysis, with every node representing a unique intervention. The nodes with the most direct interactions were between Plat and Erlo, Plat and Gef, and Osi and Plat (Figure 1B). Erlo+Bev (HR 0.26, 95% CrI: 0.08-0.75), Osi (HR 0.29, 95% CrI: 0.11-0.70), Afa (HR 0.37, 95% CrI: 0.17-0.78), and Erlo (HR 0.46, 95% CrI: 0.21-0.93) showed significant superiority over placebo in terms of PFS. Moreover, the probability for Erlo+Bev ranked first, followed by Osi, Afa, and Erlo; the ranking probabilities were 0.164, 0.164, 0.120, and 0.128, respectively (Figure 1C, 1D). The results of the comparisons among those treatments and all treatments are shown in Figure 1C and Appendix Table 4 in the Supplementary Data. The model fit was good, and there was no significant heterogeneity or loop inconsistency (Table 2).

**Table 2:** Edge-splitting method for direct and indirect evidence relating to PFS, OS, ORR and grade 3 or higher AEs in treatment-level.

**Multiple-treatment**	**Direct comparison outcome**	**Indirect comparison outcome**	**Combined outcome**	**I^2^**
**PFS** (HR and 95% CrI)				
Erlo vs Gef	0.94 (0.42-2.10)	0.84 (0.41-1.70)	0.88 (0.53-1.50)	< 50%
Afa vs Gef	0.73 (0.23-2.30)	0.71 (0.28-1.80)	0.72 (0.36-1.50)	< 50%
Dac vs Gef	0.62 (0.20-1.90)	0.97 (0.38-2.60)	0.79 (0.40-1.60)	< 50%
Osi vs Gef	0.46 (0.15-1.40)	0.65 (0.23-1.80)	0.55 (0.27-1.10)	< 50%
Plat vs Gef	1.90 (0.94-3.80)	2.10 (1.10-4.20)	2.0 (1.30-3.20)	< 50%
Placebo vs Gef	2.40 (1.20-5.30)	1.40 (0.48-3.80)	1.90 (1.10-3.60)	< 50%
Ico vs Erlo	0.78 (0.24-2.50)	1.60 (0.47-5.30)	1.10 (0.47-2.50)	< 50%
Dac vs Erlo	1.00 (0.30-3.60)	0.83 (0.30-2.40)	0.90 (0.43-2.00)	< 50%
Plat vs Erlo	2.50 (1.40-4.50)	2.00 (0.88-4.30)	2.30 (1.40-3.60)	< 50%
Plat vs Ico	1.50 (0.51-4.70)	3.10 (0.86-11.00)	2.10 (0.91-4.80)	< 50%
Plat vs Afa	3.90 (1.20-12.00)	2.20 (0.79-5.90)	2.80 (1.30-5.80)	< 50%
Plat vs Osi	3.20 (1.40-7.70)	4.60 (1.30-16.00)	3.70 (1.80-7.20)	< 50%
Placebo vs Afa	2.00 (0.58-6.70)	3.50 (1.30-11.00)	2.70 (1.30-6.00)	< 50%
Placebo vs Dac	2.1 (0.65-6.70)	2.80 (0.96-8.80)	2.40 (1.10-5.30)	< 50%
**OS** (HR and 95% CrI)				
Plat vs Gef	1.30 (0.25-7.00)	0.93 (0.32-2.60)	1.00 (0.44-2.60)	< 50%
Vin vs Gef	0.35 (0.06-2.10)	NA	0.35 (0.09-1.40)	NA
Placebo vs Gef	2.30 (0.36-14.00)	0.91 (0.26-3.20)	1.20 (0.51-4.00)	< 50%
Erlo vs Gef	0.84 (0.17-4.60)	1.10 (0.43-2.70)	1.00 (0.48-2.40)	< 50%
Dac vs Gef	0.77 (0.15-4.10)	0.99 (0.37-2.60)	0.92 (0.44-2.40)	< 50%
Osi vs Gef	0.63 (0.12-3.30)	NA	0.63 (0.20-2.00)	NA
Erlo vs Sun + Erlo	0.72 (0.13-4.00)	NA	0.72 (0.21-2.50)	NA
Cet + Plat vs Cil + Cet +Plat	1.00 (0.19-5.70)	NA	1.10 (0.32-3.50)	NA
Cet + Plat vs Cet + Bev +Plat	1.20 (0.24-6.30)	NA	1.20 (0.39-3.80)	NA
Plat vs Cet + Plat	1.40 (0.26-7.40)	NA	1.40 (0.44-4.30)	NA
Erlo vs Plat	1.20 (0.33-4.10)	0.88 (0.32-2.50)	0.99 (0.46-2.20)	< 50%
Ico vs Plat	1.00 (0.20-5.30)	NA	1.00 (0.32-3.30)	NA
Erlo vs Doc	0.38 (0.05-2.70)	NA	0.38 (0.08-1.90)	NA
Ico vs WBRT	0.93 (0.17-4.90)	NA	0.94 (0.29-3.10)	NA
Afa vs Placebo	1.60 (0.30-9.20)	NA	1.60 (0.47-5.80)	NA
Dac vs Placebo	0.98 (0.19-5.20)	0.68 (0.20-2.30)	0.77 (0.27-1.90)	< 50%
Dac vs Erlo	0.94 (0.16-5.80)	0.90 (0.30-2.70)	0.91 (0.37-2.40)	< 50%
Erlo + Bev vs Erlo	1.20 (0.19-7.20)	NA	1.2 (0.28-4.80)	NA
Ona +Erlo vs Erlo	4.60 (0.54-41.00)	NA	4.70 (0.71-30.00)	NA
Erlo + Tiv vs Erlo	0.72 (0.35-1.50)	NA	0.72 (0.20-2.60)	NA
**ORR** (OR and 95% CrI)				
Plat vs Gef	0.16 (0.03-0.72)	0.22 (0.06-0.78)	0.19 (0.07-0.49)	< 50%
Afa vs Gef	1.78 (0.39-8.07)	1.33 (0.39-4.54)	1.49 (0.56-3.78)	< 50%
Dac vs Gef	1.17 (0.26-5.23)	NA	1.19 (0.37-3.70)	NA
Gef + Peme vs Gef	1.16 (0.33-3.51)	NA	1.16 (0.42-3.00)	NA
Afa vs Plat	6.38 (1.43-29.91)	8.84 (2.60-30.11)	7.78 (3.02-20.37)	< 50%
Osi vs Plat	2.81 (0.89-7.70)	NA	2.96 (1.16-6.36)	NA
Cet + Plat vs Plat	2.08 (0.45-9.29)	NA	2.03 (0.65-6.60)	NA
Osi vs Erlo	1.26 (0.27-5.57)	NA	1.26 (0.41-3.98)	NA
Naq vs Erlo	0.53 (0.12-2.39)	NA	0,53 (0.17-1.67)	NA
Erlo + Bev vs Erlo	1.30 (0.44-3.90)	NA	1.32 (0.55-3.11)	NA
**Grade 3 or higher AEs** (OR and 95% CrI)				
Plat vs Gef	0.16 (0.03-0.72)	0.22 (0.06-0.78)	0.19 (0.07-0.49)	< 50%
Afa vs Gef	1.78 (0.39-8.07)	1.33 (0.39-4.54)	1.49 (0.56-3.78)	< 50%
Dac vs Gef	1.17 (0.26-5.23)	NA	1.19 (0.37-3.70)	NA
Gef + Peme vs Gef	1.16 (0.33-3.51)	NA	1.16 (0.42-3.00)	NA
Afa vs Plat	6.38 (1.43-29.91)	8.84 (2.60-30.11)	7.78 (3.02-20.37)	< 50%
Osi vs Plat	2.81 (0.89-7.70)	NA	2.96 (1.16-6.36)	NA
Cet + Plat vs Plat	2.08 (0.45-9.29)	NA	2.03 (0.64-6.60)	NA
Osi vs Erlo	1.26 (0.27-5.57)	NA	1.26 (0.41-3.98)	NA
Naq vs Erlo	0.53 (0.12-2.39)	NA	0.53 (0.17-1.67)	NA
Erlo + Bev vs Erlo	1.30 (0.44-3.90)	NA	1.32 (0.55-3.11)	NA

Abbreviations: PFS, progression-free survival; OS, overall survival; ORR, objective response rate; AEs, adverse events; NA, not available.

Abbreviations of available treatments could be found in the main body of manuscript.

**Figure 2:**
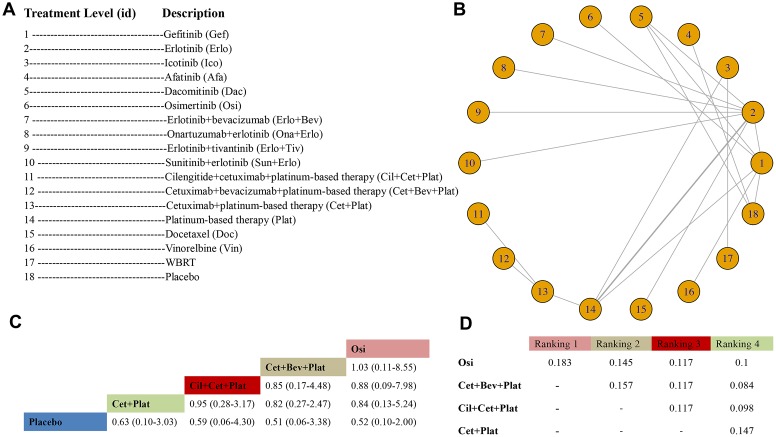
**Meta-analysis of efficacy for OS at the individual-treatment level.** (**A**) Descriptions of the individual treatments included in this analysis. (**B**) Network plots showing comparisons between nodes (yellow circles), each representing a unique individual treatment. Each line corresponds to direct comparisons between treatments, with the width corresponding to the number of direct within-trial comparisons. (**C**) Comparison results of the most efficacious treatments and placebo (HR (95% CrI)). Each result is a comparison between the column-defining drug and the row-defining treatment. (**D**) Schematic detailing the most efficacious treatments according to the rankograms.

A total of 21 RCTs with 21 arms and 18 unique treatment levels were considered in the OS analysis; a study by Reck et al. [45] was excluded from the network (Figure 2A). Eighteen nodes were included in the treatment-level analysis for OS. The most direct interactions were in nodes between Erlo and Plat (Figure 2B). Osi (HR 0.52, 95% CrI: 0.10-2.00), Cet+Bev+Plat (HR 0.51, 95% CrI: 0.06-3.38), Cil+Cet+Plat (HR 0.59, 95% CrI: 0.06-4.30), and Cet+Plat (HR 0.63, 95% CrI: 0.10-3.03) appeared to benefit OS over placebo even though the favorable efficacy was nonsignificant. Osi (ranking probability: 0.183) ranked first, followed by Cet+Bev+Plat (0.157), Cil+Cet+Plat (0.117), and Cet+Plat (0.147) (Figure 2C, 2D). The results of the comparisons among those treatments and all treatments are shown in Figure 2C and Appendix Table 4 in the Supplementary Data. The statistical model was good, and no evidence of heterogeneity or loop inconsistency was observed (Table 2). A total of 13 trials with 10 unique treatment levels were analyzed for ORR after excluding Reck et al. [45] and Yang et al. (2) [38] (Appendix Figure 2 in Supplementary Data). The primary data are shown in Appendix Table 5 in the Supplementary Data. Afa ranked first and had the best ORR (OR 7.67, 95% CrI: 2.93-20.68 vs Plat), followed by Dac (OR 6.10, 95% CrI: 1.34-29.20), Gef (OR 5.14, 95% CrI: 1.96-14.10), and Gef + Peme (OR 6.02, 95% CrI: 1.42-23.13) (Appendix Table 6 in the Supplementary Data). No evidence of heterogeneity was found. A total of 17 trials with 13 unique treatment levels focused on grade 3 or higher AEs (Appendix Figure 3 in the Supplementary Data). The primary data are shown in Appendix Table 7 in the Supplementary Data. Erlo + Bev had the most grade 3 or higher AEs (OR 24.22, 95% CrI: 0.64-NA vs Ico), followed by Gef +Peme (OR 15.28, 95% CrI: 0.43-NA), Plat (OR 12.61, 95% CrI: 0.86-NA) and Dac (OR 11.09, 95% CrI: 0.33-NA) (Appendix Table 8 in the Supplementary Data). No significant heterogeneity was noted (Table 2). The outcome of DoR was not analyzed for insufficient comparisons.

### Bayesian NMA at the medication-class level

**Figure 3:**
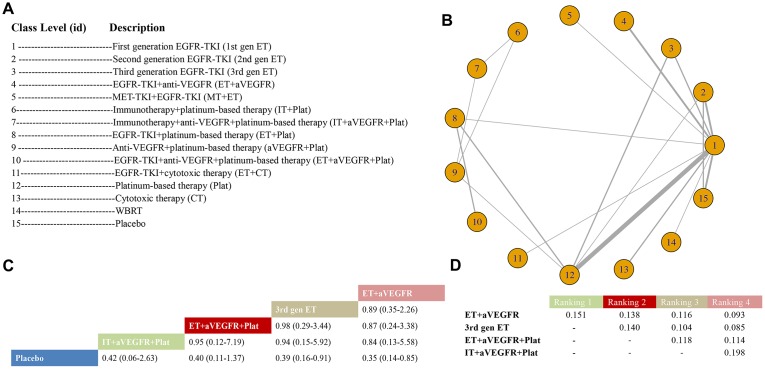
**Meta-analysis of efficacy for PFS at the medication-class level.** (**A**) Descriptions of the medication classes included in this analysis. (**B**) Network plots showing comparisons between nodes (yellow circles), each representing a unique medication class. Each line corresponds to direct comparisons between treatments, with the width corresponding to the number of direct within-trial comparisons. (**C**) Comparison results of the most efficacious class and placebo (HR (95% CrI)). Each result is a comparison between the column-defining drug and the row-defining class. (**D**) Schematic detailing the most efficacious medication classes according to the rankograms.

There were 35 RCTs with 36 arms and 15 unique class levels included in the class-level analysis for PFS; the studies of Yang et al. [39], Urata et al. [32], and Shi et al. [17] were excluded for having two of the same class interventions. A total of 15 nodes were presented, in which the most direct interventions were between 1^st^-gen ET and Plat, 1^st^-gen ET and 2^nd^-gen ET, and 1^st^-gen ET and Placebo (Figure 3A and Figure 3B). ET+aVEGFR (HR 0.35, 95% CrI: 0.14-0.85) and 3^rd^-gen ET (HR 0.39, 95% CrI: 0.16-0.91) were better than placebo, while ET+aVEGFR+Plat (HR 0.40, 95% CrI: 0.11-1.37) and IT+aVEGFR+Plat (HR 0.42, 95% CrI: 0.06-2.63) seemed to be superior than placebo, but the data were not statistically significant (Figure 3C). Our work demonstrated that statistically, ET+aVEGFR ranked first, followed by 3^rd^-gen ET, ET+aVEGFR+Plat, and IT+aVEGFR+Plat (Figure 3D). The results of the comparisons among those treatments and all treatments are shown in Figure 3C and Appendix Table 9 in the Supplementary Data. The model was good, and no heterogeneity or inconsistency was observed.

**Figure 4:**
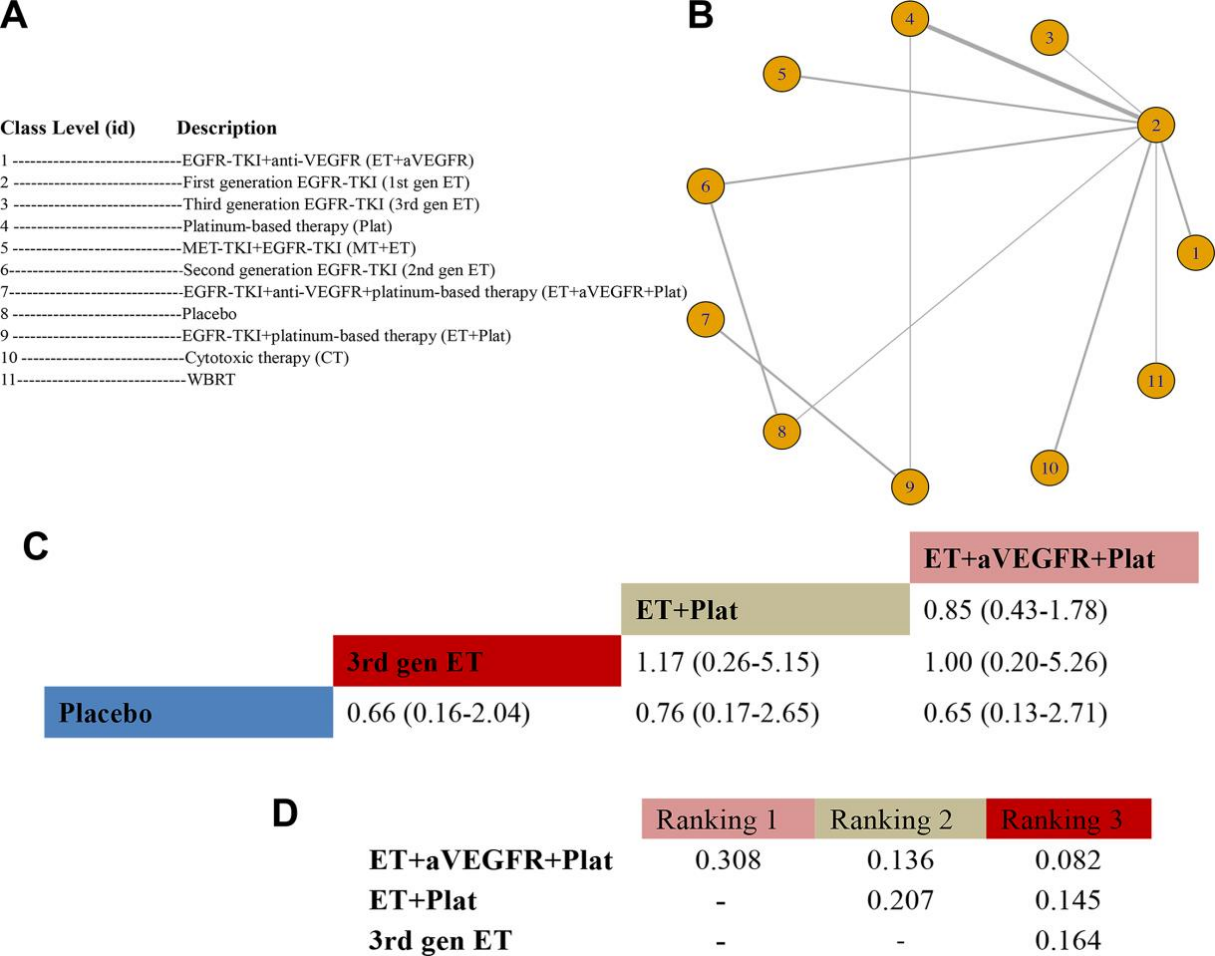
**Meta-analysis of efficacy for OS at the medication-class level.** (**A**) Descriptions of the medication classes included in this analysis. (**B**) Network plots showing comparisons between nodes (yellow circles), each representing a unique medication class. Each line corresponds to direct comparisons between treatments, with the width corresponding to the number of direct within-trial comparisons. (**C**) Comparison results of the most efficacious class and placebo (HR (95% CrI)). Each result is a comparison between the column-defining drug and the row-defining class. (**D**) Schematic detailing the most efficacious medication classes according to the rankograms.

In total, 21 RCTs with 11 arms and 11 unique class levels were selected for the class-level analysis of OS; studies by Yang et al. [39] and Reck et al. [45] were excluded for comparing the same class interventions (Figure 4A). The 11 nodes with the most direct interactions were between 1^st^-gen ET and Plat (Figure 4B). When compared with placebo, the HR for ET+aVEGFR+Plat was 0.65 (95% CrI: 0.13-2.71), the HR for ET+Plat was 0.76 (95% CrI: 0.17-2.65), and the HR for 3^rd^-gen ET was 0.66 (95% CrI: 0.16-2.04) (Figure 4C). Bayesian analysis revealed that ET+aVEGFR+Plat was significantly ranked first, followed by ET+Plat, and then 3^rd^-gen ET (Figure 4D). Additional data on the interactions can be found in Figure 4C and Appendix Table 9 in the Supplementary Data. The fitness for this model was good, and no significant heterogeneity or inconsistencies were observed.

In medication-class level analysis for ORR (Appendix Figure 4 in the Supplementary Data, Appendix Table 5 in the Supplementary Data), 2^nd^-gen ET was the best (OR 12.52, 95% CrI: 3.38-49.41 vs WBRT), followed by ET + aVEGFR (OR 11.64, 95% CrI: 2.87-49.19) and 1^st^-gen ET (OR 8.82, 95% CrI: 2.79-29.56) (Appendix Table 10 in the Supplementary Data). Analysis for DoR (Appendix Figure 5 in the Supplementary Data, Appendix Table 11 in the Supplementary Data) revealed that 2^nd^-gen ET had the longest DoR (MD 5.08, 95% CrI: -11.44 to 21.63 vs ET + aVEGFR), followed by 3^rd^-gen ET (MD 3.04, 95% CrI: -11.29 to 17.31) and ET + CT (MD 3.87, 95% CrI: -12.47 to 20.38) (Appendix Table 12 in the Supplementary Data). Analysis for grade 3 or higher AEs (Appendix Figure 6 in the Supplementary Data, Appendix Table 7 in the Supplementary Data) revealed that ET + aVEGFR had the most grade 3 or higher AEs (OR 9.01, 95% CrI: 0.99-92.27 vs Placebo), followed by Plat (OR 5.25, 95% CrI: 0.98-29.83) and ET + CT (OR 3.82, 95% CrI: 0.39-37.26) (Appendix Table 13 in the Supplementary Data). There was low to moderate heterogeneity among medication-level comparisons on ORR, DoR and grade 3 or higher AEs.

### EGFR genomic status

Multiple treatment-level comparisons (10 treatments) were specifically performed for 19 del and 21 L858R mutations regarding PFS. We found that Erlo+Bev, Osi, Afa, and Erlo were top-ranking alternatives for patients harboring 19 del mutations to prolong PFS; additionally, Erlo+Bev, Osi, Gef+Pem, and Dac were optimal treatments among the available treatments for patients with 21 L858R mutations (Table 3). Overall, Erlo+Bev, Osi, and 2^nd^-gen ET (including Afa and Dac) were the most viable treatment options, and no significant differences were found in terms of the best strategies for 19 del and 21 L858R mutations. The evidence was insufficient to assess these mutations in terms of OS as well as uncommon mutations.

**Table 3:** Comparisons of PFS for exon 19 deletion and exon 21 Leu-858Arg mutation according to treatment-level Bayesian analysis.

Gef	0.76 (0.26-2.20)	1.63 (0.27-10.40)	0.56 (0.13-2.23)	0.70 (0.22-2.23)	0.68 (0.22-2.15)	0.41 (0.09-1.80)	0.67 (0.16-2.95)	**3.19 (1.10-9.82)**	4.18 (0.41-44.40)
1.31 (0.45-3.89)	Erlo	2.15 (0.44-11.80)	0.73 (0.13-4.30)	0.92 (0.26-3.40)	0.90 (0.26-3.40)	0.54 (0.19-1.54)	0.88 (0.15-5.69)	**4.21 (1.99-9.59)**	5.49 (0.61-53.60)
0.61 (0.10-3.67)	0.47 (0.08-2.26)	Ico	0.34 (0.03-3.25)	0.43 (0.07-2.63)	0.42 (0.06-2.59)	0.25 (0.03-1.66)	0.41 (0.04-4.07)	1.96 (0.46-8.03)	2.57 (0.56-11.70)
1.80 (0.45-7.48)	1.37 (0.23-7.84)	2.95 (0.31-30.60)	Dac	1.27 (0.21-7.83)	1.23 (0.21-7.75)	0.74 (0.09-5.51)	1.21 (0.16-9.75)	**5.76 (1.91-34.90)**	7.53 (0.51-125.00)
1.43 (0.45-4.46)	1.09 (0.29-3.86)	2.34 (0.38-15.40)	0.79 (0.13-4.88)	Afa	0.97 (0.23-4.14)	0.58 (0.11-2.97)	0.95 (0.15-6.19)	**4.57 (1.46-15.10)**	5.99 (0.57-64.80)
1.47 (0.47-4.50)	1.12 (0.31-3.86)	2.41 (0.39-15.90)	0.81 (0.13-4.85)	1.03 (0.24-4.31)	Osi	0.60 (0.11-3.05)	0.99 (0.15-6.34)	**4.69 (1.50-14.70)**	6.15 (0.57-67.30)
2.47 (0.56-11.30)	1.87 (0.65-5.33)	4.06 (0.60-28.70)	1.36 (0.18-10.90)	1.73 (0.34-9.23)	1.68 (0.33-9.23)	Erlo+Bev	1.65 (0.21-14.20)	**7.90 (2.24-30.00)**	10.30 (0.92-121.00)
1.50 (0.34-6.39)	1.13 (0.18-6.72)	2.45 (0.25-25.50)	0.83 (0.10-6.24)	1.05 (0.16-6.84)	1.01 (0.16-6.54)	0.61 (0.07-4.83)	Gef+Peme	4.79 (0.76-31.20)	6.24 (0.41-100.00)
**0.31 (0.10-0.91)**	**0.24 (0.10-0.50)**	0.51 (0.13-2.19)	**0.17 (0.03-0.99)**	0.22 (0.07-0.69)	**0.21 (0.07-0.67)**	**0.13 (0.03-0.45)**	0.21 (0.03-1.31)	Plat	1.30 (0.16-10.70)
0.24 (0.02-2.47)	0.18 (0.02-1.63)	0.39 (0.09-1.79)	0.13 (0.01-1.95)	0.17 (0.02-1.76)	0.16 (0.01-1.76)	0.10 (0.01-1.09)	0.16 (0.01-2.44)	0.77 (0.09-6.16)	WBRT

Results for exon 19 deletion are shown in blue-colour cells, results for exon 21 Leu 858Arg mutation are in gray-color cells. Comparisons should be read from left to right and the estimate is in the cell in common between the column-defining drugs and the row-defining treatment. For 19 del and 21 L858R mutations, HRs (and 95% CrI) less than 1 favour the column-defining treatment. To obtain HRs for comparisons in the opposite direction, reciprocals should be taken. Significant results are in bold and underscored. Abbreviations: Gef, gefitinib; Erlo, erlotinib; Ico, icotinib; Dac, dacomitinib; Afa, afatinib; Osi, osimertinib; Erlo+Bev, erlotinib+bevacizumab; Gef+Peme, gefitinib+pemetrexed; Plat, platinum-based therapy.

### Sensitivity analysis

Sensitivity analyses helped to confirm the robustness of these findings, which put more weight on main outcomes. The results restricted to phase III trials (trial number = 34 and patient number = 7448) did not show significant deviations compared with the original network Bayesian analyses; however, they showed a more robust status of Osi that had better OS and PFS. Superiority of Erlo + Bev on PFS was also stressed. The results of the primary meta-analysis remained stable across sensitivity analyses by removing Reck et al. [45], (IMpower150) and Soria et al. [42], (FLAURA), respectively. The superiority of Osi was confirmed to be robust. When restricted to Asia (trial number = 12 and patient number = 1977), the superiority of Osi on PFS slightly declined, but Osi was still among the top-ranking treatments. Erlo + Bev and EGFR-TKI + aVEGFR + Plat showed favorable PFS across Asian and non-Asian populations (trial number = 29 and patient number = 6408).

## DISCUSSION

With increasing molecular research focused on biomarkers for NSCLC, attention has been drawn to targeted therapies and immunotherapy. The available guidelines have helped improve the level of clinical treatment, but they still need to be updated as more evidence and clinical trials emerge. In the present study, we gathered evidence from 41 RCTs assessing the role of 22 treatment-level options and 22 class-level options for the intended populations. Herein, we found that Erlo+Bev, Osi, Afa, and Erlo are optimal treatment-level options in terms of PFS and that Osi, Cet+Bev+Plat, Cil+Cet+Plat, and Cet+Plat are successively optimal options in terms of OS. For medications at the class level, ET+aVEGFR, 3^rd^-gen ET, ET+aVEGFR+Plat, and IT+aVEGFR+Plat outperform other treatments in terms of PFS, while ET+aVEGFR+Plat, followed by ET+Plat and 3^rd^-gen ET, are better alternatives in terms of OS. Additionally, Erlo+Bev and Osi were superior to other treatments for 19 del and 21 L858R mutations in terms of PFS. Although these were modest differences, most ORR and DoR results were consistent with the PFS and OS results. Erlo +Bev elicited the most severe AEs, which should be properly managed during clinical use. Regarding the efficacy, top-ranking treatments for advanced EGFR-mutant NSCLC also include first-line settings such as osimertinib, while their AEs should be considered in clinical medication. These findings provide crucial implications for clinical reference.

ET monotherapy has been established as the standard treatment for patients with EGFR-positive NSCLC, and a meta-analysis involving patients who had not previously received treatment showed a median PFS of 11 months with ET (gefitinib/erlotinib) versus 5.6 months with chemotherapy [56]. However, most patients with lung cancer are diagnosed at an advanced stage, and the prognosis remains poor despite novel therapeutics. To improve PFS, combination treatments with 1^st^-gen ET and 2^nd^-gen ET have been evaluated in several clinical trials [57]. Bev inhibits angiogenesis by restricting oxygen and nutrient supplies to suppress tumor growth, and combination chemotherapy with Bev has been shown to be effective against CNS metastasis and pleural effusion [58, 59], but the conclusion is controversial. Several clinical trials (JO25567, NEJ026) [22, 46] included in this study also compared the efficacy of Erlo+Bev with that of Erlo, and the results showed that PFS was significantly prolonged in the Erlo+Bev group. A meta-analysis [60] investigating Erlo+Bev compared with monotherapy for NSCLC found no substantial benefits for the OS or PFS of all patients, but the combination treatment significantly enhanced OS for EGFR-mutant patients. The mechanism by which Erlo+Bev improves PFS is still unclear, but there are some hypotheses. Bev might normalize blood flow, thus improving drug delivery to tumor blood vessels [61, 62]. Autocrine or paracrine signaling by the VEGF receptor might catalyze cancer cell proliferation and produce anti-apoptotic effects, which could be inhibited by Bev to restore apoptosis [63]. Resistance by Erlo+Bev to the VEGF-mediated pathway has been confirmed in basic research [64]. Previous evidence has demonstrated that the response induced by the 21 L858R mutation was inferior to that induced by the 19 del mutation, which could be improved by Erlo+Bev. However, in this study, Erlo+Bev was found to be the preferred treatment for both 21 L858R and 19 del mutations. Although the OS endpoint was or met and was not significantly different, further clinical validation is still needed. When combined with ET, Bev+Plat has shown promising efficacy among patients with liver metastasis harboring EGFR mutations, which was consistent with our findings. Accordingly, Erlo+ET+Plat also offers potential benefits for patients with advanced EGFR-mutant NSCLC.

Osi is an oral 3^rd^-gen ET that selectively inhibits ET sensitivity and EGFR T790M resistance (present in almost 60% of patients) and has been approved for the treatment of patients with metastatic T790M-positive NSCLC. Preclinical and clinical data (AURA3) [34, 65] support the ability of Osi to cross the blood-brain barrier and penetrate the CNS; the PFS of advanced EGFR-mutant NSCLC in the FLAURA trial [42] was significantly enhanced in the Osi group. Moreover, the OS of the Osi group was also significantly enhanced (38.6 months vs 31.8 months of standard ET; HR 0.48, 95% CI, 0.26-0.86), as reported by an abstract presented at the 2019 ESMO conference. Patients with T790M resistance mutations who receive Osi may still eventually exhibit progression, and thus, they require better treatment options. Reck et al. [45] noted improved survival with immunotherapy+Bev+Plat compared with Bev+Plat, which suggested the potential priority of immunotherapy in the intended populations. The randomized phase III trial CheckMate 227 [66] demonstrated that nivolumab+ipilimumab resulted in a longer OS duration than chemotherapy, regardless of the programmed cell death-ligand 1 (PD-L1) expression level. Before the further application of immunotherapy in real clinical practice, we should understand the role of tumor mutational burden (TMB) as a biomarker and note safety concerns.

This study has several clinical implications and strengths that should be mentioned. To the best of our knowledge, this study is the most comprehensive work comparing treatment effectiveness for advanced EGFR-mutant NSCLC to date. Current national and international guidelines are mostly based on the results of single RCTs, as well as standard meta-analyses dedicated to the pairwise comparisons of two or three treatments. Moreover, with the large number of treatment options, meta-analyses of direct comparisons are inevitably limited by the relatively small number of studies assessing a particular pair of treatments. Meta-analyses on multiple treatments reduce this issue by creating indirect comparisons and allowing data synthesis that helps to identify the best treatment. This study is the first to address the efficacy of therapeutics for advanced EGFR-mutant NSCLC with state-of-the art Bayesian methods. The conclusions are also strengthened by the highest level of evidence. Finally, this study is based on multivariable, time-varying HRs that assumed proportional hazards, examined the relative treatment efficacy based on parameters of survival plots (shape and scale), and considered the influence of time [67]. Network studies regarding relative risks or odds ratios do not have these strengths.

There are also several limitations that should be acknowledged. First, one or two studies were omitted from the treatment-level and medication-level analyses for not contributing to a complete network loop. Although the credibility of our conclusion might not be maximized, the omitted studies had little impact on our final results. Second, the results on ORR and DoR might not be as well reported because of the limited study number. Additionally, potential AEs could influence our judgment on the preferred treatment. Third, the OS outcome was not as comprehensively reported by the primary studies as PFS, and the results based on statistical data still need clinical validation.

## CONCLUSIONS

This is the first Bayesian NMA to show promising, significant efficacy and safety for Erlo+Bev, Erlo+Bev+Plat and Osi over other available treatments for advanced EGFR-mutant NSCLC, considering the balance between PFS and OS. Erlo+Bev and Osi are the top-ranked regimens for patients with either 19 del or 21 L858R mutations. Accordingly, the three strategies can be reasonably recommended to these intended patients based on their effectiveness. However, their AEs should also be determined in real clinical practice. IT+aVEGFR+Plat is a potential superior regimen but still needs to be confirmed by more clinical trials. The current study not only provides evidence for the use of Erlo+Bev but also addresses the landscape of the use of Osi in advanced EGFR-mutant NSCLC. In the future, more evidence is needed to develop novel therapies and to identify the best treatment options for patients according to their NSCLC subtype and for those with site-specific metastases.

## MATERIALS AND METHODS

This article was based on the Preferred Reporting Items for Systematic Reviews and Meta-Analyses extension statement for network meta-analyses of health care interventions (Appendix Table 1 in the Supplementary Data). The protocol was registered with PROSPERO (CRD42019137033).

### Search strategy

Related published trials were identified after a rigorous literature search in PubMed, EMBASE, Cochrane Library and Clinical Trials.gov from their inception to September 2019. The key terms used were “EGFR mutant”, “non-small cell lung cancer”, “NSCLC”, and “randomized controlled trials” (Appendix Material and Methods in the Supplementary Data). No language restrictions were applied. Reference lists were searched manually for additional records.

### Selection criteria

All published RCTs involving adult patients (≥18 years) whose Eastern Cooperative Oncology Group (ECOG) performance status was 0 or 1 and comparing any systematic interventions (pharmaceutical, surgical, radiological, combinations, etc.) for histologically or cytologically confirmed advanced (stage III/IV/recurrent/distant metastasis (brain, liver, bone, etc.)) EGFR-mutant NSCLC was identified. There were no mandatory restrictions on first-line treatment settings or other-line settings. The included patients within the selected trials must have positive and clear advanced EGFR-mutant cancer diagnoses. The duration period of the eligible trials was no less than 6 months. No further restrictions were applied on other individual-level (i.e., age, sex) or program-level characteristics (i.e., start year, follow-up period). If a multi-arm trial compared one treatment to two or more different treatments, we extracted every arm/comparison. The most recent and informative publication was selected to avoid duplications. We excluded trials comparing different administration schemes with the same drug or combinations. Dose-expansion trials, reviews, and fundamental experiments were also excluded.

### Definitions of outcomes and treatment arms

In this study, the primary outcomes were PFS and OS according to the Response Evaluation Criteria in Solid Tumors (RECIST version 1.1). The secondary outcomes were objective response rate (ORR), duration of response (DoR, month) and grade 3 or higher adverse events (AEs) (severe AEs). Eligible studies should report at least one of both clinical outcomes. EGFR mutations included exon 19 deletion (19 del), exon 21 Leu858Arg mutation (21 L858R) and other uncommon mutations (19 del and 21 L858R were the main focus) [47].

To organize the current treatment options in clinical trials into clinically meaningful arms, we used general prespecified criteria, as shown in Table 1. Cilengitide (Cil) and cetuximab (Cet) are seldom used in NSCLC, and for statistical convenience and network simplification, Cil is categorized in the aVEGFR class, and Cet is categorized in the ET class [48].

### Data extraction and quality assessment

Relevant data were independently extracted by two investigators following our prespecified protocol. Any discrepancies were resolved by discussion with a third investigator. The extracted information included characteristics of the eligible trials (publication year, first author, trial name, follow-up period, number of arms, etc.), characteristics of the populations (mean age, number of enrolled patients, etc.), and characteristics of the program (types of systematic interventions, outcomes of intended endpoints, registration information, etc.). Outcome estimates were extracted using fully adjusted models. Additionally, we contacted the authors if there were any missing data. If we received no response, the analysis was performed without these data. Intent-to-treat data were used when available.

The risk of bias of the included RCTs was assessed using the modified Cochrane Collaboration’s risk of bias tool [49]. The two coauthors performed a quality assessment on all the included RCTs. In the case of disagreements, the two authors rechecked the original articles, and a consensus was achieved after a discussion.

### Statistical analysis

For PFS and OS, the hazard ratios (HRs) and confidence intervals (CIs) were directly extracted from the original studies or were calculated by methods provided by Tierney et al. [50]. We also tried to contact the authors if the study provided only figures without exact data. If the authors did not respond, the program Engauge Digitizer 4.1 (http://digitizer.sourceforge.net) was run to extract the exact data from the figures. Odd ratios (ORs) for ORR and grade 3 or higher AEs were manually calculated based on extracted information.

A Bayesian network meta-analysis (NMA) was performed with a random effects model to estimate the HR and 95% credible interval (95% CrI) for direct and indirect evidence on advanced EGFR-mutant NSCLC by combining multiple systematic arms across studies with all the information regarding PFS and OS. In the case of multi-arm trials (trials with three or more interventions), adjustments were made to preserve randomization and correlation within the multi-arm trials by converting log-HRs to log-hazards. ORs and 95% CrI in the random effects model were prepared for ORR and grade 3 or higher AEs for direct and indirect evidence; the mean difference (MD) and 95% CrI in random effects was conducted for DoR because DoR was regarded as a continuous variable. Following the Cochrane Handbook [49], the standard deviation (SD) was roughly computed by the (Xmax-Xmin)/range difference for further analysis.

The Markov chain Monte Carlo (MCMC) method was used to estimate the posterior distribution of each parameter, and the fit of the random effects model was assessed by the deviance information criteria (DIC) [51, 52]. A hierarchical Bayesian model synthesizes comparisons between the treatment pairs and simultaneously summarizes all outcomes of interest by assuming a common heterogeneity parameter (a derived I^2^ statistic > 50% or a P value for Cochran Q chi-square test <0.1 was regarded as indicating significant heterogeneity) [53, 54]; the inconsistency of this model was evaluated by the edge-splitting method based on all direct and indirect evidence [54]. To confirm the robustness of our findings, sensitivity analyses were performed with studies restricted to phase III trials, studies excluding Reck et al. [45] and Soria et al. [42], respectively and Asian and non-Asian studies. The relative treatment rankings were graphically displayed with rankograms [55].

In the Bayesian context, the statistical significance of HRs and ORs was established when the 95% CrI did not contain 1, and that of MDs was established when it did not contain 0. Calculations were performed in R version 3.5.3 (www.r-project.org) using the gemtc and rjags packages, which are publicly available. The detailed statistical methods are provided in the Appendix Material and Methods in the Supplementary Data.

### Data availability statement

Data sharing is not applicable to this article as no new data were created or analyzed in this study.

## Supplementary Material

Supplementary Materials and Methods

Appendix Figures

Appendix Table 1

Appendix Table 2

Appendix Tables 3, 6-13

Appendix Table 4

Appendix Table 5
