# Supplementary material for: Therapeutic options for advanced epidermal growth factor receptor (EGFR)-mutant non-small cell lung cancer: a Bayesian network secondary analysis
Source: Aging (Albany NY). 2020 Apr 23;12(8):7129–62. doi: 10.18632/aging.103066 (PMC7202525; doi:10.18632/aging.103066)
Supplement: Supplementary Materials and Methods [file aging-12-103066-s005..pdf]

SUPPLEMENTARY MATERIALS

Supplementary Materials and Methods

This article was according to the Preferred Reporting Items for Systematic Reviews and Meta-Analyses extension statement for network meta-analysis for health care. The protocol was registered with PROSPERO (CRD42019137033).

Search strategy

Relating published trials were identified after a rigorous literature search on PubMed, EMBASE, Cochrane Library and Clinical Trials.gov from inception to Sep 2019. The key items used were "EGFR mutant", "EGFR mutation", "non-small cell lung cancer", "NSCLC", "randomized controlled trials". No restrictions were applied on language. Reference lists were searched manually for additional records [1, 2].

Comprehensive searches were conducted in four electronic databases:

(1) PubMed/Medline (NLM)
(2) EMBASE (Elsevier)
(3) Cochrane Library (CENTRAL/Wiley)
(4) Clinical Trials.gov (NIH)

The literature search strategy was developed first in PubMed and then translated to the other databases. A combination of relevant keywords and controlled vocabulary (MeSH - Medical Subject Headings in PubMed and Emtree in EMBASE) were used in the PubMed and EMBASE searches. Comparable keyword search strategies were used in Cochrane Central Register of Controlled Trials (CENTRAL) and Clinical Trials.gov [3].

No date or language restrictions were applied. Results were limited to Human clinical trials. MEDLINE records were excluded from EMBASE results sets.

Four component concepts made up the search strategy:

(1) NSCLC
(2) EGFR-mutant
(3) Advanced cancer
(4) RCTs

Before total search, we used the Cochrane Childhood Cancer Group search strategy for *Cancer* in PubMed: (and adapted it to the other databases) for more restrictions and precision.(cancer OR cancers OR cancer* OR oncology OR oncolog* OR neoplasm OR neoplasms OR neoplasm* OR carcinoma OR carcinom* OR tumor OR tumour OR tumor* OR tumour* OR tumors OR tumours OR malignan* OR malignant).

For search set #4, we used Cochrane Handbook recommended search filters for finding RCTs.

Search filters were used for finding RCTs in PubMed and EMBASE. Available database limiters were used in Cochrane CENTRAL (Trials)

http://work.cochrane.org/pubmed
sensitivity- and precision-maximizing version (2008 revision); PubMed format

(randomized controlled trial[pt] OR controlled clinical trial[pt] OR randomized[tiab] OR placebo[tiab] OR clinical trials as topic[mesh:noexp] OR randomly[tiab] OR trial[ti] NOT (animals[mh] NOT humans [mh])

http://work.cochrane.org/embase
Embase search strategy for finding RCTs in Embase

('crossover procedure':de OR 'double-blind procedure':de OR 'randomized controlled trial':de OR 'single-blind procedure':de OR (random* OR factorial* OR crossover* OR cross NEXT/1 over* OR placebo* OR doubl* NEAR/1 blind* OR singl* NEAR/1 blind* OR assign* OR allocat* OR volunteer*):de,ab,ti)

Each of the four components of the search strategy was first searched upon individually, combining synonyms describing that concept with the Boolean operator OR. The four individual component search sets were then combined together using the Boolean operator AND.

Resulting citations were managed and duplicates removed using the Endnote citation management software program X8 (Thomson Reuters).

	PubMed/MEDLINE Search Strategy
**1 non-small cell lung cancer (NSCLC)**	("Carcinoma, non-small cell lung"[Mesh] OR "carcinomas, non-small cell lung" OR "lung carcinoma, non-small cell" OR " lung carcinomas, non-small cell " OR "non-small cell lung carcinomas" OR "nonsmall cell lung cancer" OR "non-small-cell lung carcinoma" OR "non small cell lung carcinoma" OR "carcinoma, non-small cell lung " OR "non-small cell lung cancer")
**2 EGFR mutant**	("EGFR" OR "EGFR-mutant" OR "EGFR mutations" OR "egfr" OR "aerobic capacity" OR " epidermal growth factor receptor" OR

	"EGFR-mutant patients" OR "patients with EGFR mutations")
**3 Advanced**	("Advanced" OR "terminal" OR "late" OR "late stage" OR " stage IIIA " OR " stage IIIB" OR " stage III" OR " stage IV" OR "metastasis" OR "recurrent" OR "recurrence")
**4 RCT**	(randomized controlled trial[pt] OR controlled clinical trial[pt] OR randomized[tiab] OR placebo[tiab] OR clinical trials as topic[mesh:noexp] OR randomly[tiab] OR trial[ti])
**5**	(animals[mh] NOT humans [mh])
**6**	1 AND 2 AND 3 AND 4
**7**	6 NOT 5

Abbreviations: Mesh = Medical Subject Heading, pt = Publication Type, tiab = Title/Abstract, ti = Title, mh = MeSH Terms.

Selection criteria

All the published RCTs of adult patients (≥18 year) whose ECOG status was 0 or 1 that compared any systematic interventions (pharmaceutical, surgical, radiological, combinations etc.) for advanced EGFR-mutant NSCLC were identified. No mandatory restrictions on first-line treatment settings or other-line settings. The included patients within selected trials must have positive and clear advanced EGFR mutant cancer diagnoses. Duration period of eligible trials should not be less than 1 year. No further restrictions were applied on other individual-level and program-level characteristics. If a multi-arm trial compared one treatment to two or more different treatments, we extracted every arm/comparison respectively. The most recent and informative publication was selected for avoiding duplication. We excluded trials comparing different administration schemes with the same drug or combinations. Dose-expansion trials, reviews, fundamental experiments were also excluded.

Definitions of outcomes and treatment arms

In this study, the primary outcomes were PFS and overall survival (OS) according to the Response Evaluation Criteria in Solid Tumors (RECIST version 1.1). The secondary outcomes were objective response rate (ORR), duration of response (DoR, month) and grade 3 or higher adverse events (AEs) (severe AEs). Eligible studies should report at least one of the both clinical outcomes. EGFR mutations include regular exon 19 deletion (19 del) and exon 21 Leu858Arg mutation (21 L858R) and other uncommon mutations (19 del and 21 L858R were mainly focused).

To organize the current treatment options took in clinical trials into clinically meaningfully arms, we used general prespecified criteria. Systematic treatments in the study were summarized as treatment level and medication class level. Treatment level included: gefitinib (Gef), erlotinib (Erlo), icotinib (Ico), afatinib (Afa), dacomitinib (Dac), osimertinib (Osi), naquotinib (Naq), erlotinib+bevacizumab (Erlo+Bev), onartuzumab +erlotinib (Ona+Erlo), erlotinib+tivantinib (Erlo+Tiv), sunitinib+erlotinib (Sun+Erlo), gefitinib+pemetrexed (Gef+Peme), cilengitide+cetuximab+platinum-based therapy (Cil+Cet+Plat), cetuximab+bevacizumab+ platinum-based therapy (Cet+Bev+Plat), cetuximab+ platinum-based therapy (Cet+Plat), erlotinib+platinum-based therapy (Erlo+Plat), motesanib+platinum-based therapy (Mot+Plat), platinum-based therapy (Plat), docetaxel (Doc), vinorelbine (Vin), whole-brain radiotherapy (WBRT) and placebo.

Medication class level included: first generation EGFR-TKI (1st-gen ET), second generation EGFR-TKI (2nd-gen ET), third generation EGFR-TKI (3rd-gen ET), EGFR-TKI+anti-VEGFR (ET+aVEGFR), MET-TKI+ EGFR-TKI (MT+ET), immunotherapy+platinum-based therapy (IT+Plat), immunotherapy+anti-VEGFR+ platinum-based therapy (IT+aVEGFR+Plat), EGFR-TKI+platinum-based therapy (ET+Plat), anti-VEGFR+ platinum-based therapy (aVEGFR+Plat), EGFR-TKI+anti-VEGFR+platinum-based therapy (ET+aVEGFR +Plat), EGFR-TKI+anti-VEGFR+platinum-based therapy (ET+aVEGFR+Plat), EGFR-TKI+cytotoxic therapy (ET+ CT), platinum-based therapy (Plat), cytotoxic therapy (CT), whole-brain radiotherapy (WBRT), and placebo.

Actually, cilengitide and cetuximab were rarely used in NSCLC, for the statistical convenience and the network simplification, cilengitide was regarded as aVEGFR class and cetuximab was outlined into ET class.

Data extraction and quality assessment

Relevant data were extracted by two independent investigators following our prespecified protocol. Any discrepancies would be resolved by a discussion with a third investigator. The extracted information included: characteristics of the eligible trials (publication year, the first author, trial name, follow-up period, number of arms etc.), characteristics of the populations (mean age, number of enrolled patients etc.), characteristics of the program (types of systematic interventions, outcomes of intended endpoints, registration information etc.). Outcome estimates were extracted in fully adjusted models. Additionally, we contacted the authors if there were any miss data. If we received no response, analysis was performed without these data. Intent-to-treat data were used when available.

Risk of bias of included RCTs was assessed using the modified Cochrane Collaboration's Risk-of-Bias Tool [3]. Two coauthors performed quality assessment on all the included RCTs. The Cochrane Risk of Bias Tool was adopted to assess risk of bias for each RCT. Seven items were used to evaluate heterogeneity in each trial: randomization sequence generation, allocation concealment, blinding of participants and personnel, blinding of outcome assessment, incomplete outcome data, selective reporting, and other biases. The quality of each study was categorized as high, low, or unclear. In case of disagreement, the two authors would recheck the original articles and a consensus would be achieved after a discussion.

Statistical analysis

For PFS and OS, the hazard ratios (HR) and confidence intervals (CI) were directly extracted from the original studies or were calculated by methods provided by Tierney et al [4]. We also tried to contact the authors if the study provided only figures without exact data. In case the authors did not respond, the program Engauage Digitizer 4.1 (http://digitizer.sourceforge.net) was run to extract the exact data from the figures. This program can calculate clear values by digitizing data points from an image file after the manual setting of the coordinate axis. Odd ratios (ORs) for ORR and grade 3 or higher AEs were manually calculated based on extracted information.

A Bayesian network meta-analysis (NMA) was performed with a random effects model to estimate the HR and 95% credible interval (95% CrI) for direct and indirect evidence on advanced EGFR-mutant NSCLC by combining multiple systematic arms across studies with all the information regarding PFS and OS. In the case of multi-arm trials (trials with three or more interventions), adjustments were made to preserve randomization and correlation within the multi-arm trials by converting log-HRs to log-hazards. ORs and 95% CrI in random effects model were prepared for ORR and grade 3 or higher AEs for direct and indirect evidence; mean difference (MD) and 95% CrI in random effects was conducted for DoR, because DoR was a continuous variable. Following Cochrane Handbook, standard deviation (SD) was roughly computed by (Xmax-Xmin)/range difference for further analysis. The Markov Chain Monte Carlo (MCMC) method was used to estimate the posterior distribution of each parameter, the fit of the random effects model was assessed by the deviance information criteria (DIC) [5–8]. A three-chain model with non-informative priors was run with an adaptation phase of 10000 iterations followed by 100000 model iterations. The thin ratio was set to 10. Non-convergence was assessed by the Gelman-Rubin statistic. Relative treatment rankings (probability for each treatment to be the most effective (first best regime), the second best, the third best and so on) were displayed graphically with rankograms, which indicated the probable best and worst therapies [9]. A hierarchical Bayesian model synthesizes comparisons between the treatment pairs and simultaneously summarizes all outcomes of interest by assuming a common heterogeneity parameter (a derived I^2 statistic > 50% or a P value for Cochran Q chi-square test <0.1 was regarded as indicating significant heterogeneity), the inconsistency of this model was evaluated by the edge-splitting method based on all direct and indirect evidence [3, 8]. Trace, density and comsol estimations/plots were used to inspect the uncertainty of the MCMC model [5–8]. To confirm the robustness of our findings, sensitivity analyses were performed restricted on phase III trials, studies excluding Reck et al, 2019 and excluded Soria et al, 2018 respectively and Asian and non-Asian.

In the Bayesian context, statistical significance of HRs and ORs was established when the 95% CrI did not contain 1, of MDs was established when did not contain 0. Calculations were performed in R version 3.5.3 (https://www.r-project.org) using the gemtc and jag etc. public packages.

Supplementary References

1. Herbst RS, Morgensztern D, Boshoff C. The biology and management of non-small cell lung cancer. Nature. 2018; 553:446–54. https://doi.org/10.1038/nature25183 PMID:29364287

2. Cedra S, Wiegand S, Kolb M, Dietz A, Wichmann G. Reduced Cytokine Release in Ex Vivo Response to Cilengitide and Cetuximab Is a Marker for Improved Survival of Head and Neck Cancer Patients. Cancers (Basel). 2017; 9:E117. https://doi.org/10.3390/cancers9090117 PMID:28872582

3. Higgins JP, Altman DG, Gøtzsche PC, Jüni P, Moher D, Oxman AD, Savovic J, Schulz KF, Weeks L, Sterne JA, and Cochrane Bias Methods Group, and Cochrane Statistical Methods Group. The Cochrane Collaboration's tool for assessing risk of bias in randomised trials. BMJ. 2011; 343:d5928. https://doi.org/10.1136/bmj.d5928 PMID:22008217

4. Tierney JF, Stewart LA, Ghersi D, Burdett S, Sydes MR. Practical methods for incorporating summary time-to-event data into meta-analysis. Trials. 2007; 8:16. https://doi.org/10.1186/1745-6215-8-16 PMID:17555582

5. Caldwell DM, Ades AE, Higgins JP. Simultaneous comparison of multiple treatments: combining direct and indirect evidence. BMJ. 2005; 331:897–900. https://doi.org/10.1136/bmj.331.7521.897 PMID:16223826

6. Mavridis D, Salanti G. A practical introduction to multivariate meta-analysis. Stat Methods Med Res. 2013; 22:133–58. https://doi.org/10.1177/0962280211432219 PMID:22275379

7. Lu G, Ades AE. Combination of direct and indirect evidence in mixed treatment comparisons. Stat Med. 2004; 23:3105–24. https://doi.org/10.1002/sim.1875 PMID:15449338

8. Higgins JP, Thompson SG, Deeks JJ, Altman DG. Measuring inconsistency in meta-analyses. BMJ. 2003; 327:557–60. https://doi.org/10.1136/bmj.327.7414.557 PMID:12958120

9. Salanti G, Ades AE, Ioannidis JP. Graphical methods and numerical summaries for presenting results from multiple-treatment meta-analysis: an overview and tutorial. J Clin Epidemiol. 2011; 64:163–71. https://doi.org/10.1016/j.jclinepi.2010.03.016 PMID:20688472
