# Supplementary material for: Therapeutic options for advanced epidermal growth factor receptor (EGFR)-mutant non-small cell lung cancer: a Bayesian network secondary analysis
Source: Aging (Albany NY). 2020 Apr 23;12(8):7129–62. doi: 10.18632/aging.103066 (PMC7202525; doi:10.18632/aging.103066)
Supplement: Appendix Figures [file aging-12-103066-s007..pdf]

APPENDIX FIGURES

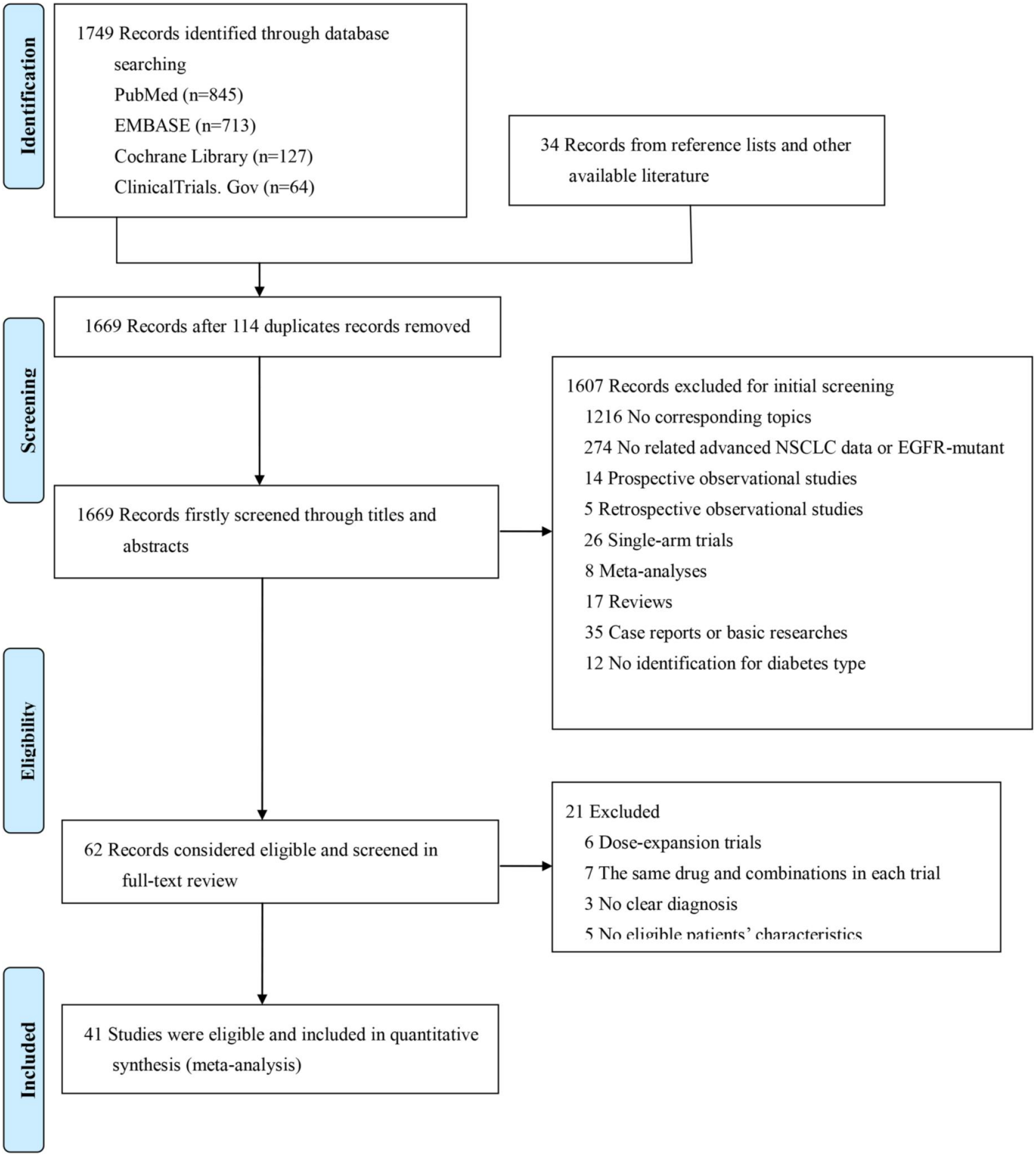

Appendix Figure 1. Study flow chart.

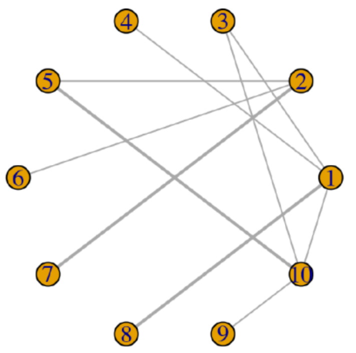

1 = Gef   2 = Erlo   3 = Afa   4 = Dac   5 = Osi   6 = Naq   7 = Erlo + Bev   8 = Gef + Peme   9 = Cet + Plat   10 = Plat

Appendix Figure 2. Network plot for ORR in treatment-level analysis.

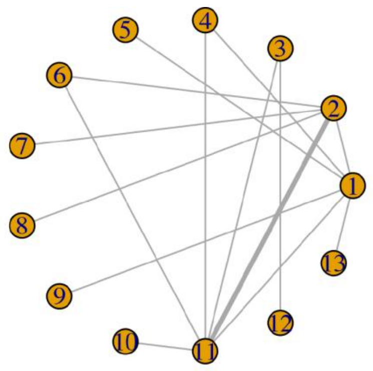

1 = Gef   2 = Erlo   3 = Ico   4 = Afa   5 = Dac   6 = Osi   7 = Naq

8 = Erlo + Bev   9 = Gef + Peme   10 = Cet + Plat   11 = Plat

12 = WBRT   13 = Placebo

Appendix Figure 3. Network plot for grade 3 or higher AEs in treatment-level analysis.

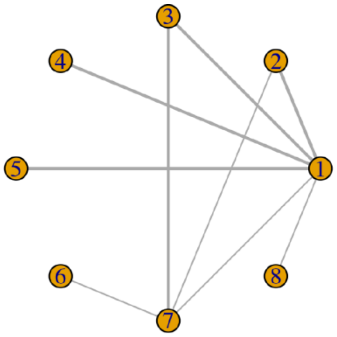

1 = 1st gen ET   2 = 2nd gen ET   3 = 3rd gen ET   4 = ET + aVEGFR

5 = ET + CT   6 = ET + Plat   7 = Plat   8 = WBRT

Appendix Figure 4. Network plot for ORR in class-level analysis.

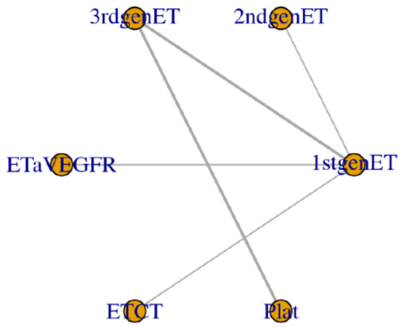

Treatments include: **1st gen ET**, **2nd gen ET**, **3rd gen ET**, **ET + aVEGFR**, **ET + CT**, **Plat**

Appendix Figure 5. Network plot for DoR in class-level analysis.

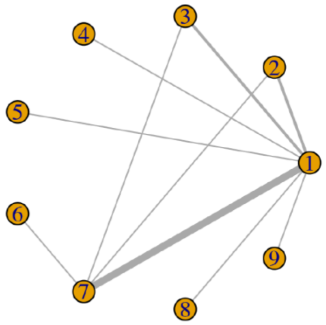

1 = 1st gen ET   2 = 2nd gen ET   3 = 3rd gen ET   4 = ET + aVEGFR

5 = ET + CT   6 = ET + Plat   7 = Plat   8 = WBRT   9 = Placebo

Appendix Figure 6. Network plot for grade 3 or higher AEs in class-level analysis.
