# Supplementary material for: Therapeutic options for advanced epidermal growth factor receptor (EGFR)-mutant non-small cell lung cancer: a Bayesian network secondary analysis
Source: Aging (Albany NY). 2020 Apr 23;12(8):7129–62. doi: 10.18632/aging.103066 (PMC7202525; doi:10.18632/aging.103066)
Supplement: Appendix Table 2 [file aging-12-103066-s001..docx]

**Appendix Table A2**. Baseline characteristics of included studies in this Bayesian study

| Study | Mean age | Metastasis (number) | period | Intervention arm (number) | Control arm (number) | Outcome (HR, 95% CI) | Trial name (registration information) | EGFR genomic aberration data (HR, 95% CI)‡ |
| --- | --- | --- | --- | --- | --- | --- | --- | --- |
| Saito et al, 2019^46^ (Japan) | 67.5 y | CNS metastasis (72) | 2015.6-2017.12 | erlotinib + bevacizumab (112) | erlotinib (112) | **PFS**: 0.61 (0.42-0.88) | NEJ026 (UMIN000017069) | **19 del** 56/55 PFS: 0.69 (0.41-1.16); **21 L858R** 56/57 PFS: 0.57 (0.33-0.97) |
| Reck et al, 2019^45^ (multiple nations)* | NA | NA | 2015.3-2018.1 | atezolizumab+bevacizumab+carboplatin and paclitaxel (34) | bevacizumab+carboplatin and paclitaxel (45) | **PFS**: 0.61 (0.36-1.03); **OS**: 0.61 (0.29-1.28) | IMpower150 (NCT02366143) |  |
|  |  |  |  | atezolizumab+carboplatin and paclitaxel (45) | bevacizumab+carboplatin and paclitaxel (45) | **PFS**: 1.14 (0.73-1.78); **OS**: 0.93 (0.51-1.68) |  |  |
| Kelly et al, 2019^44^ (America) | 67.5 y | NA | 2016.2-2017.12 | naquotinib(267) | gefitinib/erlotinib (263) | **PFS**: 1.61 (1.09-2.39) | NCT02588261 |  |
| Wu et al, 2018^43^ (multiple nations) | 58.4 y | CNS metastasis (116) | 2014.8-2016.8 | osimertinib (75) | platinum-pemetrexed (41) | **PFS**: 0.32 (0.15-0.69) | AURA3 (NCT02151981) |  |
| Soria et al, 2018^42^ (multiple nations) | 64.0 y | CNS metastasis (116); Visceral metastasis (197) | 2014.12-2016.3 | osimertinib (279) | gefitinib/erlotinib (277) | **PFS**: 0.46 (0.37-0.57); **OS**: 0.63 (0.45-0.88) | FLAURA (NCT02296125) | **19 del** 175/174 PFS: 0.43 (0.32-0.56), 21 **L858R** 104/103 PFS: 0.51 (0.36-0.71) |
| Mok et al, 2018^41^ (multiple nations) | 61.5 y | NA | 2013.4-2016.7 | dacomitinib (227) | gefitinib (225) | **OS**: 0.76 (0.58-0.99) | ARCHER 1050 (NCT01774721) | **19 del** 134/133 OS: 0.88 (0.61-1.26); 21 **L858R** 93/92 OS: 0.71 (0.48-1.05) |
| Herbst et al, 2018^40^ (multiple nations) | 63.0 y | NA | 2009.8-2014.5 | cetuximab+paclitaxel+carboplatin+bevacizumab (199) | cetuximab+paclitaxel+carboplatin (201) | **PFS**: 0.92 (0.75-1.12); **OS**: 0.81 (0.66-1.00) | SWOG S0819 (NCT00946712) |  |
| Yang et al, 2017^39^ (China) | NA | CNS metastasis (47) | 2009.7-2014.7 | erlotinib (128) | gefitinib (128) | **PFS**: 0.81 (0.62-1.05); **OS**: 0.84 (0.63-1.13) | CTONG 0901 (NCT01024413) |  |
| Yang et al (2), 2017^38^ (China) | 57.5 y | CNS metastasis (158) | 2012.12-2015.6 | icotinib (85) | WBRT (73) | **PFS**: 0.56 (0.36-0.90); **OS**: 0.93 (0.60-1.44) | BRAIN (NCT01724801) | **19 del** 45/48 PFS: 0.39 (0.20-0.75); **21 L858R** 36/21 PFS: 0.79 (0.37-1.67) |
| Wu et al, 2017^37^ (multiple nations) | 61.5 y | NA | 2013.5-2015.3 | dacomitinib (227) | gefitinib (225) | **PFS**: 0.62 (0.50-0.78); | ARCHER 1050 (NCT01774721) | **19 del** 134/133 PFS: 0.55 (0.41-0.75); **21 L858R** 93/92 PFS: 0.63 (0.44-0.88) |
| Spigel et al, 2017^36^ (multiple nations) | NA | NA | 2012.1-2013.8 | onartuzumab+erlotinib (28) | erlotinib (29) | **PFS**: 1.15 (0.48-2.76); **OS**: 4.68 (0.97-22.63) | METLung (NCT01456325) |  |
| Shi et al, 2017^35^ (China) | 56.0 y | CNS metastasis (81) | 2013.1-2014.8 | icotinib (148) | cisplatin+pemetrexed (137) | **PFS**: 0.65 (0.48-0.88); **OS**: 1.03 (0.69-1.52) | CONVINCE | **19 del** 80/74 PFS: 0.51 (0.34-0.76) **21 L858R** 68/63 PFS: 0.90 (0.60-1.35); |
| Mok et al, 2017^34^ (multiple nations) | 62.3 y | CNS metastasis (144); visceral metastasis (225) | 2014.8-2015.9 | osimertinib (279) | pemetrexed+carboplatin/cisplatin (140) | **PFS**: 0.30 (0.23-0.41); | AURA3 (NCT02151981) | **19 del** 191/87 PFS: 0.34 (0.24-0.46);21 **L858R** 83/45 PFS: 0.46 (0.30-0.71) |
| Kubota et al, 2017^33^ (multiple nations) | NA | NA | 2012.7-2015.3 | motesanib+paclitaxel+carboplatin (41) | paclitaxel+carboplatin (42) | **PFS**: 0.57 (0.33-0.98) | AMG-706 (JPRN-JapicCTI- 121887) |  |
| Urata et al, 2016^32^ (multiple nations) | 67.5 y | NA | 2009.7-2012.10 | erlotinib (198) | gefitinib (203) | **PFS**: 1.09 (0.88-1.36) | WJOG 5108L ( | **19 del** 102/90 PFS: 1.12 (0.81-1.54); **21 L858R** 80/92 PFS: 0.94 (0.68-1.30) |
| Park et al, 2016^31^ (multiple nations) | 63.0 y | adrenal glands (28); bone (153); liver (40); lung ipsilateral (174); lung contralateral (138); other (204) | 2011.12-2013.8 | afatinib (160) | gefitinib (159) | **PFS**: 0.73 (0.57-0.95); | LUX-Lung 7 (NCT01466660) | **19 del** 93/93 PFS: 0.76 (0.55-1.06); **21 L858R** 67/66 PFS: 0.71 (0.48-1.06) |
| Cheng et al, 2016^30^ (multiple nations) | 61.7 y | NA | 2012.2-2013.8 | gefitinib+pemetrexed (126) | gefitinib (65) | **PFS**: 0.68 (0.48-0.96) | (NCT01469000) | **19 del** 65/40 PFS: 0.67 (0.43-1.05); **21 L858R** 52/23 PFS: 0.58 (0.33-1.01) |
| Zhou et al, 2015^29^ (China) | NA | NA | 2008.8-2012.7 | erlotinib (82) | gemcitabine+carboplatin (72) | **OS**: 1.19 (0.83-1.71) | CTONG-0802 (NCT00874419) | **19 del** 43/39 OS: 1.52 (0.92-2.52); **21 L858R** 39/33 OS: 0.92 (0.55-1.54) |
| Wu et al, 2015^28^ (China) | 56.8 y | NA | 2011.3-2012.6 | erlotinib (110) | gemcitabine/cisplatin (107) | **PFS**: 0.34 (0.22-0.51) | ENSURE (NCT01342965) | **19 del** 57/61 PFS: 0.20 (0.11-0.37); **21 L858R** 52/46 PFS: 0.57 (0.31-1.05) |
| Vansteenkiste et al, 2015^27^ (multiple nations) | NA | NA | 2009.2-2013.7 | cilengitide+cetuximab+platinum-based chemotherapy (48) | cetuximab+platinum-based chemotherapy (44) | **PFS**: 0.57 (0.32-0.99); **OS**: 0.95 (0.56-1.61) | CERTO (NCT00842712) |  |
| Soria et al, 2015^26^ (multiple nations) | 59.0 y | CNS metastasis (75); other (243) | 2012.3-2013.12 | gefitinib (133) | placebo (132) | **PFS**: 0.86 (0.65-1.13) | IMPRESS (NCT01544179) |  |
| Scagliotti et al, 2015^25^ (multiple nations) | NA | NA | 2011.1-2012.7 | erlotinib+tivantinib (56) | erlotinib (53) | **OS**: 0.72 (0.35-1.48) | ARQ 197 (NCT01244191) |  |
| Yang et al, 2014^24^ (multiple nations) | NA | NA | 2009.12-2012.8 | pemetrexed+cisplatin+gefitinib (26) | gefitinib (24) | **PFS**: 0.83 (0.42-1.62) | NCT01017874 |  |
| Wu et al, 2014^23^ (multiple nations) | 58.0 y | NA | 2010.8-2011.11 | afatinib (242) | cisplatin+gemcitabine (122) | **PFS**: 0.26 (0.19-0.36) | LUX-Lung 6 (NCT01121393) | **19 del** 124/62 PFS: 0.20 (0.13-0.33); **21 L858R** 92/46 PFS: 0.32 (0.19-0.52) |
| Seto et al, 2014^22^ (Japan) | 67.0 y | NA | 2011.2-2012.3 | erlotinib+bevacizumab (75) | erlotinib (77) | **PFS**: 0.54 (0.36- 0.79); **OS**: 1.16 (0.45-3.00) | JO25567 (JapicCTI-111390) | **19 del** 40/40 PFS: 0.41 (0.24-0.72); **21 L858R** 35/37 PFS: 0.67 (0.38-1.18) |
| Ramalingam et al, 2014^21^ (multiple nations) | NA | NA | 2011.6-2013.3 | dacomitinib (47) | erlotinib (44) | **PFS**: 1.04 (0.58-1.85); **OS**: 0.93 (0.39-2.19) | ARCHER 1009 (NCT01360554) |  |
| Kawaguchi et al, 2014^20^ (Japan) | NA | NA | 2009.8-2012.7 | erlotinib (21) | docetaxel (30) | **PFS**: 0.82 (0.43-1.53); **OS**: 0.38 (0.10-1.10) | DELTA |  |
| Ellis et al, 2014^19^ (multiple nations) | NA | NA | 2009.12-2013.6 | dacomitinib (114) | placebo (68) | **PFS**: 0.48 (0.35-0.66); **OS**: 0.98 (0.67-1.44) | NCIC CTG BR.26 (NCT01000025) |  |
| Wu et al, 2013^18^ (multiple nations) | NA | NA | 2009.4-2010.9 | erlotinib+gemcitabine+carboplatin/cisplatin (49) | gemcitabine+carboplatin/cisplatin (48) | **PFS**: 0.25 (0.14-0.44) | FASTACT-2 (NCT00883779) |  |
| Shi et al, 2013^17^ (China) | NA | NA | 2009.2-2009.12 | icotinib (29) | erlotinib (39) | **PFS**: 0.78 (0.47-1.28) | ICOGEN (ChiCTR-TRC-09000506) |  |
| Zhang et al, 2012^16^ (China) | 53.0 y | NA | 2008.9-2009.8 | gefitinib (15) | placebo (15) | **PFS**: 0.17 (0.07-0.42) | (INFORM; C-TONG 0804 (NCT00770588) |  |
| Scagliotti et al, 2012^15^ (multiple nations) | NA | NA | 2007.8-2009.2 | sunitinib+erlotinib (28) | erlotinib (30) | **PFS**: 1.19 (0.62-2.30); **OS**: 1.39 (0.76-2.57) | NA |  |
| Rosell et al, 2012^14^ (multiple nations) | 63.8 y | CNS metastasis (20); bone metastasis (57) | 2007.2-2011.1 | erlotinib (86) | cisplatin+docetaxel/gemcitabine (87) | **PFS**: 0.37 (0.25-0.54) | EURTAC (NCT00446225) | **19 del** 57/58 PFS: 0.30 (0.18-0.50); **21 L858R** 29/29 PFS: 0.55 (0.29-1.02) |
| Pirker et al, 2012^13^ (multiple nations) | 60.5 y | NA | 2007.2-2012.12 | cetuximab+cisplatin+vinorelbine (178) | cisplatin+vinorelbine (167) | **PFS**: 0.86 (0.68-1.09); **OS**: 0.73 (0.58-0.93) | FLEX (NCT00148798) |  |
| Miller et al, 2012^12^ (multipke nations) | NA | NA | 2008.5-2009.9 | afatinib (62) | placebo (34) | **PFS**: 0.51 (0.31-0.85); **OS**: 1.65 (0.89-3.04) | LUX-Lung 1 (NCT00656136) |  |
| Gridelli et al, 2012^11^ (multiple nations) | NA | NA | 2006.12-2009.11 | erlotinib (19) | cisplatin+gemcitabine (20) | **PFS**: 1.71 (0.82-3.60); **OS**: 1.13 (0.32-3.96) | TORCH (NCT00349219) |  |
| Zhou et al, 2011^10^ (China) | 57.9 y | NA | 2008.8-2009.7 | erlotinib (82) | gemcitabine+carboplatin (72) | **PFS**: 0.16 (0.10-0.26) | OPTIMAL (NCT00874419) | **19 del** PFS: 0.13 (0.07-0.25); **21 L858R** PFS: 0.26 (0.14-0.49) |
| Maemondo et al, 2010^9^ (Japan) | 63.3 y | NA | 2006.3-2009.5 | gefitinib (114) | carboplatin+paclitaxel (114) | **PFS**: 0.30 (0.22-0.41); **OS**: 0.75 (0.49-1.17) | (UMIN-CTR number, C000000376) |  |
| Mok et al, 2009^8^ (multiple natiions) | NA | NA | 2006.3-2007.10 | gefitinib (132) | carboplatin+paclitaxel (129) | **PFS**: 0.48 (0.36-0.64) | IPASS (NCT00322452) |  |
| Goss et al, 2009^7^ (multiple nations) | NA | NA | 2004.9-2006.12 | gefitinib (12) | placebo (20) | **PFS**: 0.29 (0.11-0.73); **OS**: 0.44 (0.17-1.12) | INSTEP (NCT00259064) |  |
| Crino et al, 2008^6^ (multiple nations) | 74.0 y | NA | 2004.7-2005.12 | gefitinib (30) | vinorelbine (24) | **PFS**: 3.13 (1.45-6.76); **OS**: 2.88 (1.21-6.83) | INVITE (NCT00256711) |  |
| Reference list number of each include study is consistent with the main body text and has been presented in the main body text.  * This trial has three arms that were reported respectively in the table; ‡ EGFR common mutations include exon 19 deletion and exon 21 Leu858Arg mutation. Abbreviations: EGFR, epidermal growth factor receptor; HR, hazard ratio; CI, confidence interval; OS, overall survival; PFS, progression-free survival; CNS, central nerve system; WBRT, whole brain radiotherapy; NA, not available. | | | | | | | | |
|  |  |  |  |  |  |  |  |  |
|  |  |  |  |  |  |  |  |  |
|  |  |  |  |  |  |  |  |  |
