# Supplementary material for: Therapeutic options for advanced epidermal growth factor receptor (EGFR)-mutant non-small cell lung cancer: a Bayesian network secondary analysis
Source: Aging (Albany NY). 2020 Apr 23;12(8):7129–62. doi: 10.18632/aging.103066 (PMC7202525; doi:10.18632/aging.103066)
Supplement: Appendix Tables 3, 6-13 [file aging-12-103066-s006..pdf]

APPENDIX TABLES

Appendix Table 3. Cochrane Risk of Bias for the Included Studies.

	Randomization sequence generation (selection bias)	Allocation concealment (selection bias)	Blinding of participants and personnel (performance bias)	Blinding of outcome assessment (detection bias)	Incomplete outcome data (attrition bias)	Selective reporting (reporting bias)	Other bias
**Crino, 2008[6]**	+	+	?	?	+	+	+
**Goss, 2009[7]**	+	+	?	?	+	+	+
**Mok , 2009[8]**	+	+	-	-	+	+	+
**Maemondo, 2010[9]**	+	+	+	+	+	+	+
**Zhou, 2011[10]**	+	+	-	-	+	+	?
**Gridelli, 2012[11]**	+	+	-	-	+	+	+
**Miller, 2012[12]**	+	+	+	+	+	?	+
**Pirker, 2012[13]**	+	+	-	-	+	+	?
**Rosell, 2012[14]**	+	+	-	-	+	+	+
**Scagliotti, 2012[15]**	+	+	+	+	+	+	?
**Zhang, 2012[16]**	+	+	+	+	+	+	+
**Shi, 2013[17]**	+	+	+	+	?	+	+
**Wu, 2013[18]**	+	+	+	+	+	+	+
**Ellis, 2014[19]**	+	+	+	+	+	+	?
**Kawaguchi, 2014[20]**	+	+	-	-	+	+	?
**Ramalingam, 2014[21]**	+	+	+	+	+	+	+
**Seto, 2014[22]**	+	+	-	-	+	?	?
**Wu, 2014[23]**	+	+	-	-	+	+	+
**Yang, 2014[24]**	+	+	-	-	+	?	-

**Scagliotti, 2015[25]**	+	+	+	+	+	+	+
**Soria, 2015[26]**	+	+	?	?	+	+	?
**Vansteenkiste , 2015[27]**	+	+	-	-	+	+	?
**Wu, 2015[28]**	+	+	-	-	+	?	?
**Zhou, 2015[29]**	+	+	-	-	+	+	?
**Cheng, 2016[30]**	+	+	-	-	+	+	+
**Park, 2016[31]**	+	+	-	-	+	+	?
**Urata, 2016[32]**	+	+	-	-	+	+	+
**Kubota, 2017[33]**	+	+	+	+	+	+	+
**Mok, 2017[34]**	+	+	-	-	+	+	+
**Shi, 2017[35]**	+	+	-	-	+	?	?
**Spigel, 2017[36]**	+	+	+	+	+	+	+
**Wu, 2017[37]**	+	+	-	-	+	+	+
**Yang (2), 2017[38]**	+	+	-	-	+	+	?
**Yang, 2017[39]**	+	+	?	?	+	+	?
**Herbst, 2018[40]**	+	+	-	-	+	+	+
**Mok, 2018[41]**	+	+	-	-	+	+	?
**Soria, 2018[42]**	+	+	+	+	+	+	+
**Wu, 2018[43]**	+	+	-	-	+	+	+
**Kelly, 2019[44]**	+	+	-	-	+	+	?
**Reck, 2019[45]**	+	+	-	-	+	+	+
**Saito, 2019[46]**	+	+	-	-	?	+	-

'+', yes; '-', no; '?', unclear.

Appendix Table 6. ORR of all available interventions according to treatment-level Bayesian network analysis.

Gef									
2.22 (0.44-14.61)	Erlo								
0.67 (0.25-1.80)	0.30 (0.05-1.56)	Afa							
0.84 (0.26-2.71)	0.38 (0.04-2.79)	1.26 (0.28-5.73)	Dac						
1.76 (0.53-7.49)	0.79 (0.24-2.53)	2.62 (0.79-10.86)	2.07 (0.40-13.87)	Osi					
4.14 (0.57-39.21)	1.87 (0.59-6.08)	6.22 (0.86-57.60)	4.90 (0.50-64.65)	2.37 (0.46-12.50)	Naq				
1.68 (0.27-13.68)	0.76 (0.31-1.82)	2.50 (0.40-20.46)	1.99 (0.23-22.56)	0.95 (0.23-4.17)	0.40 (0.09-1.77)	Erlo + Bev			
0.86 (0.34-2.44)	0.39 (0.05-2.74)	1.29 (0.34-5.32)	1.01 (0.23-5.02)	0.49 (0.09-2.37)	0.21 (0.02-1.98)	0.51 (0.05-4.30)	Gef + Peme		
2.52 (0.56-11.83)	1.13 (0.16-6.68)	3.77 (0.81-17.54)	3.01 (0.44-21.60)	1.44 (0.30-5.72)	0.61 (0.06-4.92)	1.51 (0.17-10.61)	2.94 (0.45-17.49)	Cet + Plat	
**5.14 (1.96-14.10)**	2.32 (0.48-8.90)	7.67 (2.93-20.68)	6.10 (1.34-29.20)	2.93 (1.10-6.37)	1.25 (0.17-7.18)	3.06 (0.50-14.90)	6.02 (1.42-23.13)	2.04 (0.62-6.58)	Plat

Note: Results for objective response rate (ORR) are shown in blue-colour cells. Comparisons should be read from left to right and the estimate is in the cell in common between the column-defining drugs and the row-defining treatment. ORs (and 95% CrI) more than 1 favour the column-defining treatment (the column-defining treatment has superior ORR). To obtain ORs for comparisons in the opposite direction, reciprocals should be taken. Significant results are in bold and underscored.
Abbreviations: Gef: gefitinib; Erlo: erlotinib; Dac: dacomitinib; Afa: afatinib; Osi: osimertinib; Naq: naquotinib; Erlo+Bev: erlotinib+bevacizumab; Gef+Peme: gefitinib+pemetrexed; Cet+Plat: cetuximab+platinum-based therapy; Plat: platinum-based therapy.

Appendix Table 7. Results of eligible studies on grade 3 or higher AEs.

Study	Intervention	Control	AEs in intervention arm	AEs in control arm	OR value
**Saito et al, 2019[46] (Japan)**	erlotinib + bevacizumab (112)	erlotinib (112)	88% (98/112)	46% (53/114)	8.06 (4.12-15.75)
**Kelly et al, 2019[44] (America)**	naquotinib (267)	gefitinib/erlotinib (263)	46% (122/267)	25.6% (67/262)	2.45 (1.70-3.54)
**Soria et al, 2018[42] (multiple nations)**	osimertinib (279)	gefitinib/erlotinib (277)	32% (89/279)	41% (114/271)	0.65 (0.46-0.91)
**Yang et al, 2017[39] (China)**	erlotinib (128)	gefitinib (128)	0% (0/128)	4.6% (6/128)	0.16 (0.02-1.35)
**Yang et al (2), 2017[38] (China)**	icotinib (85)	WBRT (73)	8% (7/85)	38% (28/73)	0.14 (0.06-0.36)
**Wu et al, 2017[37] (multiple nations)**	dacomitinib (227)	gefitinib (225)	63% (143/227)	41% (92/224)	2.44 (1.67-3.57)
**Shi et al, 2017[35] (China)**	icotinib (148)	cisplatin+pemetrexed (137)	0.7 % (1/148)	5.8% (8/137)	0.11 (0.01-1.89)
**Mok et al, 2017[34] (multiple nations)**	osimertinib (279)	pemetrexed+carboplatin/ cisplatin (140)	9% (34/279)	53.7% (73/136)	0.12 (0.07-0.20)
**Park et al, 2016[31] (multiple nations)**	afatinib (160)	gefitinib (159)	31% (50/160)	18% (29/159)	2.04 (1.21-3.44)
**Cheng et al, 2016[30] (multiple nations)**	gefitinib+pemetrexed (126)	gefitinib (65)	42% (53/126)	19% (12/65)	3.21 (1.56-6.58)
**Wu et al, 2015[28] (China)**	erlotinib (110)	gemcitabine/cisplatin (107)	40% (44/110)	56.7% (59/104)	0.51 (0.30-0.88)
**Soria et al, 2015[26] (multiple nations)**	gefitinib (133)	placebo (132)	45% (59/132)	47% (55/132)	1.13 (0.70-1.84)
**Wu et al, 2014[23] (multiple nations)**	afatinib (242)	cisplatin+gemcitabine (122)	36% (86/239)	60.2% (68/113)	0.37 (0.23-0.59)
**Rosell et al, 2012[14] (multiple nations)**	erlotinib (86)	cisplatin+docetaxel/ gemcitabine (87)	45% (38/84)	67% (55/82)	0.41 (0.22-0.76)
**Pirker et al, 2012[13] (multiple nations)**	cetuximab+cisplatin+ vinorelbine (178)	cisplatin+vinorelbine (167)	87% (153/175)	90% (151/168)	0.78 (0.40-1.53)
**Zhou et al, 2011[10] (China)**	erlotinib (82)	gemcitabine+carboplatin (72)	17% (14/83)	65% (47/72)	0.11 (0.05-0.23)
**Maemondo et al, 2010[9] (Japan)**	gefitinib (114)	carboplatin+paclitaxel (114)	41.2% (47/114)	71.7% (81/113)	0.28 (0.16-0.48)

Abbreviation: AE: adverse events.

Appendix Table 8. Grade 3 or higher AEs of all available interventions according to treatment-level Bayesian network analysis.

Gef												
1.52 (0.41-9.54)	Erlo											
4.52 (0.24-NA)	2.83 (0.15-107.80)	Ico										
0.67 (0.15-3.61)	0.45 (0.06-2.28)	0.15 (0.003-3.16)	Afa									
0.41 (0.06-2.99)	0.27 (0.02-2.57)	0.09 (0.001-3.00)	0.61 (0.04-6.93)	Dac								
2.58 (0.45-23.67)	1.71 (0.37-7.30)	0.59 (0.01-13.45)	3.81 (0.53-36.59)	6.32 (0.50-130.80)	Osi							
0.72 (0.07-12.15)	0.48 (0.07-3.43)	0.16 (0.003-5.75)	1.06 (0.09-19.10)	1.75 (0.09-56.96)	0.28 (0.02-3.42)	Naq						
0.18 (0.02-3.13)	**0.12 (0.02-0.94)**	0.04 (0.001-1.56)	0.27 (0.02-4.92)	0.45 (0.02-14.84)	**0.07 (0.005-0.92)**	0.25 (0.01-4.46)	Erlo + Bev					
0.30 (0.04-2.33)	0.20 (0.01-2.03)	0.07 (0.001-2.35)	0.44 (0.03-5.54)	0.74 (0.04-12.85)	0.12 (0.005-1.60)	0.42 (0.01-8.40)	1.64 (0.05-34.07)	Gef + Peme				
0.44 (0.04-6.42)	0.29 (0.03-2.66)	0.10 (0.002-2.93)	0.66 (0.06-9.74)	1.08 (0.05-31.43)	0.17 (0.01-2.06)	0.62 (0.03-11.77)	2.44 (0.10-46.58)	1.47 (0.07-46.40)	Cet + Plat			
0.34 (0.10-1.68)	**0.23 (0.07-0.58)**	0.08 (0.002-1.16)	0.51 (0.12-2.63)	0.84 (0.09-11.47)	**0.13 (0.03-0.56)**	0.48 (0.05-4.03)	1.88 (0.17-17.01)	1.15 (0.11-16.61)	0.77 (0.10-5.88)	Plat		
0.64 (0.02-51.08)	0.40 (0.01-25.27)	0.14 (0.02-1.12)	0.93 (0.02-76.56)	1.57 (0.02-192.40)	0.24 (0.005-17.54)	0.86 (0.01-78.54)	3.41 (0.05-NA)	2.12 (0.03-NA)	1.40 (0.03-132.70)	1.81 (0.05-106.70)	WBRT	
1.13 (0.15-8.31)	0.75 (0.04-7.33)	0.25 (0.01-8.52)	1.68 (0.12-19.88)	2.78 (0.17-47.19)	0.44 (0.02-5.78)	1.58 (0.05-30.47)	6.21 (0.18-128.00)	3.76 (0.22-68.63)	2.57 (0.09-52.73)	3.31 (0.24-32.34)	1.77 (0.01-107.60)	Placebo

Note: Results for grade 3 or higher adverse events (AEs) in individual-treatment level are shown in blue-colour cells. Comparisons should be read from left to right and the estimate is in the cell in common between the column-defining drugs and the row-defining treatment. ORs (and 95% CrI) less than 1 favour the column-defining treatment, which shows the column-defining treatment has less grade 3 or higher AEs. To obtain ORs for comparisons in the opposite direction, reciprocals should be taken. Significant results are in bold and underscored.

Abbreviations: Gef: gefitinib; Erlo: erlotinib; Ico: icotinib; Dac: dacomitinib; Afa: afatinib; Osi: osimertinib; Naq: naquotinib; Erlo+Bev: erlotinib+bevacizumab; Gef+Peme: gefitinib+pemetrexed; Cet+Plat: cetuximab+platinum-based therapy; Plat: platinum-based therapy; WBRT: whole-brain radiotherapy; NA: not available.

Appendix Table 9. PFS and OS of all available interventions according to class-level Bayesian network analysis.

1st-gen ET	0.96 (0.54-2.14)	0.63 (0.24-1.68)	1.30 (0.56-2.98)	1.14 (0.47-3.43)	NA	NA	0.74 (0.24-2.20)	NA	0.63 (0.18-2.38)	NA	1.01 (0.58-1.72)	0.77 (0.30-2.09)	1.07 (0.39-2.97)	0.97 (0.48-2.50)
1.25 (0.73-2.07)	2nd-gen ET	0.66 (0.18-1.92)	1.34 (0.41-3.58)	1.17 (0.38-3.90)	NA	NA	0.77 (0.19-2.47)	NA	0.66 (0.14-2.59)	NA	1.05 (0.39-2.24)	0.78 (0.24-2.48)	1.12 (0.29-3.40)	1.01 (0.52-2.00)
1.27 (0.69-2.32)	1.02 (0.47-2.26)	3rd-gen ET	2.06 (0.56-7.47)	1.79 (0.52-8.36)	NA	NA	1.17 (0.26-5.15)	NA	1.00 (0.20-5.26)	NA	1.59 (0.52-4.90)	1.20 (0.33-5.08)	1.71 (0.42-7.03)	1.52 (0.49-6.31)
1.42 (0.71-2.83)	1.14 (0.49-2.75)	1.12 (0.44-2.82)	ET+aVEGFR	0.88 (0.26-3.56)	NA	NA	0.56 (0.15-2.26)	NA	0.48 (0.11-2.31)	NA	0.77 (0.29-2.10)	0.59 (0.18-2.23)	0.83 (0.22-3.08)	0.75 (0.26-2.75)
0.87 (0.21-3.51)	0.70 (0.16-3.09)	0.68 (0.15-3.18)	0.62 (0.13-2.88)	MT+ET	NA	NA	0.66 (0.13-2.40)	NA	0.56 (0.10-2.46)	NA	0.89 (0.26-2.41)	0.67 (0.17-2.46)	0.95 (0.20-3.42)	0.86 (0.24-2.98)
0.63 (0.11-3.65)	0.51 (0.08-3.09)	0.50 (0.08-3.13)	0.45 (0.07-2.95)	0.73 (0.08-7.14)	IT+Plat	NA	NA	NA	NA	NA	NA	NA	NA	NA
1.19 (0.20-6.93)	0.95 (0.15-5.94)	0.94 (0.15-5.92)	0.84 (0.13-5.58)	1.36 (0.15-13.40)	1.87 (0.53-6.70)	IT+aVEGFR+Plat	NA	NA	NA	NA	NA	NA	NA	NA
0.92 (0.44-1.97)	0.74 (0.31-1.88)	0.73 (0.30-1.88)	0.65 (0.24-1.85)	1.07 (0.22-5.26)	1.45 (0.24-9.65)	0.77 (0.12-5.08)	ET+Plat	NA	0.85 (0.43-1.78)	NA	1.37 (0.53-3.58)	1.03 (0.26-4.72)	1.46 (0.33-6.42)	1.31 (0.38-5.88)
0.73 (0.20-2.64)	0.58 (0.15-2.31)	0.57 (0.15-2.28)	0.51 (0.12-2.21)	0.84 (0.13-5.74)	1.14 (0.35-3.77)	0.61 (0.18-2.04)	0.79 (0.19-3.16)	aVEGFR+Plat	NA	NA	NA	NA	NA	NA
1.24 (0.42-3.85)	1.00 (0.30-3.44)	0.87 (0.24-3.38)	1.44 (0.24-8.64)	1.96 (0.27-15.20)	1.05 (0.14-8.14)	1.35 (0.59-3.07)	1.71 (0.34-9.10)	0.84 (0.17-4.34)	ET+aVEGFR+Plat	NA	1.60 (0.48-5.10)	1.20 (0.26-6.20)	1.70 (0.32-8.68)	1.54 (0.37-7.99)
1.46 (0.47-4.73)	1.17 (0.34-4.28)	1.16 (0.32-4.41)	1.04 (0.28-4.09)	1.69 (0.28-10.60)	2.32 (0.28-19.00)	1.24 (0.15-10.40)	1.60 (0.40-6.39)	2.02 (0.36-11.40)	1.19 (0.23-5.84)	ET+CT	NA	NA	NA	NA
**0.41 (0.28-0.61)**	**0.33 (0.19-0.62)**	**0.33 (0.18-0.61)**	**0.29 (0.13-0.65)**	0.48 (0.11-2.06)	0.65 (0.12-3.63)	0.35 (0.06-1.98)	**0.45 (0.22-0.90)**	0.57 (0.17-1.94)	**0.33 (0.11-0.97)**	**0.28 (0.08-0.94)**	Plat	0.76 (0.27-2.43)	1.07 (0.34-3.40)	0.97 (0.41-2.89)
1.53 (0.61-3.87)	1.23 (0.43-3.57)	1.21 (0.41-3.67)	1.08 (0.34-3.48)	1.77 (0.33-9.37)	2.42 (0.34-17.60)	1.29 (0.18-9.49)	1.66 (0.50-5.38)	2.11 (0.43-10.30)	1.23 (0.29-5.19)	1.04 (0.23-4.53)	3.69 (1.34-10.01)	CT	1.41 (0.33-5.34)	1.29 (0.38-4.70)
0.56 (0.17-1.85)	0.45 (0.13-1.68)	0.44 (0.12-1.67)	0.40 (0.10-1.59)	0.65 (0.11-4.08)	0.88 (0.11-7.50)	0.47 (0.06-4.04)	0.61 (0.15-2.47)	0.77 (0.14-4.51)	0.45 (0.09-2.24)	0.38 (0.07-2.00)	1.35 (0.40-4.75)	1.13 (0.31-4.46)	WBRT	0.90 (0.27-3.77)
**0.50 (0.27-0.89)**	**0.40 (0.21-0.73)**	**0.39 (0.16-0.91)**	**0.35 (0.14-0.85)**	0.57 (0.12-2.59)	0.79 (0.12-4.90)	0.42 (0.06-2.63)	0.54 (0.20-1.36)	0.69 (0.16-2.72)	0.40 (0.11-1.37)	0.34 (0.09-1.19)	1.20 (0.58-1.19)	**0.32 (0.10-0.95)**	0.88 (0.22-3.27)	Placebo

Note: Results for PFS are shown in blue-colour cells, results for OS are in gray-color cells. Comparisons should be read from left to right and the estimate is in the cell in common between the column-defining drugs and the row-defining treatment. For PFS and OS, HRs (and 95% CI) less than 1 favour the column-defining treatment. To obtain HRs for comparisons in the opposite direction, reciprocals should be taken. Significant results are in bold and underscored.

Abbreviations: 1st-gen ET: first generation EGFR-TKI; 2nd-gen ET, second generation EGFR-TKI; 3rd-gen ET: third generation EGFR-TKI; ET+aVEGFR: EGFR-TKI+anti-VEGFR; MT+ET: MET-TKI+EGFR-TKI; IT+Plat: immunotherapy+platinum-based therapy;

IT+aVEGFR+Plat: immunotherapy+anti-VEGFR+platinum-based therapy; ET+Plat: EGFR-TKI+platinum-based therapy; aVEGFR+Plat: anti-VEGFR+platinum-based therapy; ET+aVEGFR+Plat: EGFR-TKI+anti-VEGFR+platinum-based therapy; ET+CT: EGFR-TKI+cytotoxic therapy; Plat: platinum-based therapy; CT: cytotoxic therapy: WBRT: whole-brain radiotherapy; PFS: progression-free survival; OS: overall survival; HR: hazard-ratio; CI: confidence interval.

Appendix Table 10. ORR of all available interventions according to class-level Bayesian network analysis.

1st-gen ET							
0.71 (0.37-1.33)	2nd-gen ET						
1.38 (0.78-2.59)	1.96 (0.92-4.57)	3rd-gen ET					
0.76 (0.35-1.66)	1.08 (0.40-2.99)	0.55 (0.20-2.99)	ET + aVEGFR				
0.86 (0.35-2.21)	1.22 (0.42-3.90)	0.62 (0.21-1.87)	1.13 (0.34-3.91)	ET + CT			
2.27 (0.66-7.47)	3.22 (0.89-11.32)	1.64 (0.47-5.21)	2.99 (0.69-12.45)	2.66 (0.54-11.41)	ET + Plat		
**4.65 (2.42-8.79)**	**6.60 (3.14-13.78)**	**3.35 (1.70-6.09)**	**6.14 (2.19-16.81)**	**5.44 (1.67-15.96)**	2.06 (0.73-5.81)	Plat	
**8.82 (2.79-29.56)**	**12.52 (3.38-49.41)**	**6.33 (1.69-24.30)**	**11.64 (2.87-49.19)**	**10.28 (2.28-45.82)**	3.89 (0.74-21.81)	1.90 (0.52-7.58)	WBRT

Note: Results for objective response rate (ORR) are shown in blue-colour cells. Comparisons should be read from left to right and the estimate is in the cell in common between the column-defining drugs and the row-defining treatment. ORs (and 95% CrI) more than 1 favour the column-defining treatment (the column-defining treatment has superior ORR). To obtain ORs for comparisons in the opposite direction, reciprocals should be taken. Significant results are in bold and underscored.
Abbreviations: 1st-gen ET: first generation EGFR-TKI; 2nd-gen ET: second generation EGFR-TKI; 3rd-gen ET: third generation EGFR-TKI; ET+aVEGFR: EGFR-TKI+anti-VEGFR; ET+CT: EGFR-TKI+cytotoxic therapy; ET+Plat: EGFR-TKI+platinum-based therapy; Plat: platinum-based therapy; WBRT: whole brain radiotherapy.

Appendix Table 11. Results of eligible studies on DoR.

Study	Intervention	Control	DoR of intervention arm (Mons)	DoR of control arm (Mons)
Saito et al, 2019[46] (Japan)	erlotinib + bevacizumab (112)	erlotinib (112)	13.5 (0.2–26.9)	12.1 (1.43–24.5)
Reck et al, 2019[45] (multiple nations)	atezolizumab+bevacizumab+carboplatin and paclitaxel (34)	atezolizumab+carboplatin and paclitaxel (45)	11·1 (2·8–18·0)	5·6 (2·6–15·2)
		bevacizumab+carboplatin and paclitaxel (45)		4·7 (2·6–13·5)
Kelly et al, 2019[44] (America)	naquotinib (267)	gefitinib/erlotinib (263)	9.17 (5.45-NA)	9.03 (7.39-NA)
Wu et al, 2018[43] (multiple nations)	osimertinib (75)	platinum-pemetrexed (41)	8.9 (4.3-NA)	5.7 (4.4-5.7)
Soria et al, 2018[42] (multiple nations)	osimertinib (279)	gefitinib/erlotinib (277)	17.2 (13.8–22.0)	8.5 (7.3–9.8)
Wu et al, 2017[37] (multiple nations)	dacomitinib (227)	gefitinib (225)	14·8 (12·0–17·4)	8·3 (7·4–9·2)
Mok et al, 2017[34] (multiple nations)	osimertinib (279)	pemetrexed+carboplatin/cisplatin (140)	9.7 (8.3-11.6)	4.1 (3.0-5.6)
Cheng et al, 2016[30] (multiple nations)	gefitinib+pemetrexed (126)	gefitinib (65)	16.2 (12.6-18.7)	10.9 (9.7-12.8)

Abbreviations: DoR: duration of response; NA: not available.

Appendix Table 12. DoR of all available interventions according to class-level Bayesian network analysis.

1st-gen ET					
-6.48 (-18.19 to 5.16)	2nd-gen ET				
-4.43 (-12.62 to 3.80)	2.09 (-12.16 to 16.42)	3rd-gen ET			
-1.36 (-13.18 to 10.23)	5.08 (-11.44 to 21.63)	3.04 (-11.29 to 17.31)	ET + aVEGFR		
-0.04 (-11.68 to 11.60)	6.46 (-9.97 to 22.94)	4.40 (-3.79 to 12.65)	1.36 (-15.13 to 17.78)	Plat	
-5.28 (-16.81 to 6.27)	1.22 (-15.28 to 17.66)	-0.84 (-15.06 to 13.50)	-3.87 (-20.38 to 12.47)	-5.25 (-21.64 to 11.28)	ET + CT

Note: Results for Duration of response (DoR) are shown in blue-colour cells. Comparisons should be read from left to right and the estimate is in the cell in common between the column-defining drugs and the row-defining treatment. MDs (Mean Difference, MD and 95% CrI) more than 0 favour the column-defining treatment (the column-defining treatment has superior DoR). To obtain MDs for comparisons in the opposite direction, reciprocals should be taken. Significant results are in bold and underscored.

Abbreviations: 1st-gen ET: first generation EGFR-TKI; 2nd-gen ET: second generation EGFR-TKI; 3rd-gen ET: third generation EGFR-TKI; ET+aVEGFR: EGFR-TKI+anti-VEGFR; ET+CT: EGFR-TKI+cytotoxic therapy; Plat: platinum-based therapy.

Appendix Table 13. Grade 3 or higher AEs of all available interventions according to class-level Bayesian network analysis.

1st-gen ET								
0.49 (0.19-1.23)	2nd-gen ET							
1.09 (0.43-2.75)	2.24 (0.63-8.12)	3rd-gen ET						
**0.12 (0.02-0.61)**	0.25 (0.04-1.61)	**0.11 (0.02-0.72)**	ET + aVEGFR					
0.30 (0.06-1.57)	0.61 (0.09-4.15)	0.27 (0.04-1.84)	2.48 (0.25-26.12)	ET + CT				
0.28 (0.05-1.60)	0.57 (0.08-3.85)	0.25 (0.04-1.73)	2.33 (0.21-25.33)	0.94 (0.08-10.14)	ET + Plat			
**0.22 (0.11-0.41)**	0.45 (0.16-1.20)	**0.20 (0.07-0.53)**	1.81 (0.31-10.43)	0.73 (0.12-4.15)	0.78 (0.15-4.00)	Plat		
**0.14 (0.02-0.77)**	0.28 (0.04-2.04)	**0.13 (0.02-0.89)**	1.14 (0.10-12.42)	0.46 (0.04-4.87)	0.49 (0.04-5.91)	0.63 (0.10-4.16)	WBRT	
1.14 (0.24-5.60)	2.34 (0.38-14.82)	1.05 (0.17-6.52)	9.51 (1.00-92.27)	3.82 (0.39-37.26)	4.07 (0.39-45.12)	5.25 (0.98-29.83)	8.36 (0.81-87.95)	Placebo

Note: Results for grade 3 or higher adverse events (AEs) are shown in blue-colour cells. Comparisons should be read from left to right and the estimate is in the cell in common between the column-defining drugs and the row-defining treatment. ORs (and 95% CrI) less than 1 favour the column-defining treatment (the column-defining treatment has less grade 3 or higher AEs. To obtain ORs for comparisons in the opposite direction, reciprocals should be taken. Significant results are in bold and underscored.

Abbreviations: 1st-gen ET: first generation EGFR-TKI; 2nd-gen ET: second generation EGFR-TKI; 3rd-gen ET: third generation EGFR-TKI; ET+aVEGFR: EGFR-TKI+anti-VEGFR; ET+CT: EGFR-TKI+cytotoxic therapy; ET+Plat: EGFR-TKI+platinum-based therapy; Plat: platinum-based therapy; WBRT: whole brain radiotherapy.
