# Supplementary material for: Therapeutic options for advanced epidermal growth factor receptor (EGFR)-mutant non-small cell lung cancer: a Bayesian network secondary analysis
Source: Aging (Albany NY). 2020 Apr 23;12(8):7129–62. doi: 10.18632/aging.103066 (PMC7202525; doi:10.18632/aging.103066)
Supplement: Appendix Table 4 [file aging-12-103066-s004..doc]

**Appendix Table A4.** Efficacy of all available interventions according to treatment-level Bayesian network analysis

| Gef | 1.02 (0.48-2.36) | 1.07 (0.26-4.64) | 2.01 (0.48-11.80) | 0.93 (0.45-2.36) | 0.63 (0.20-1.98) | NA | 1.18 (0.24-6.17) | 4.73 (0.64-36.30) | 0.74 (0.17-3.39) | 1.42 (0.34-6.37) | NA | 0.72 (0.11-4.89) | 0.61 (0.10-3.98) | 0.75 (0.18-3.28) | NA | NA | 1.03 (0.44-2.57) | 2.75 (0.47-15.90) | 0.35 (0.09-1.39) | 1.14 (0.18-7.51) | 1.22 (0.52-3.92) |
| --- | --- | --- | --- | --- | --- | --- | --- | --- | --- | --- | --- | --- | --- | --- | --- | --- | --- | --- | --- | --- | --- |
| 1.13 (0.68-1.90) | Erlo | 1.04 (0.25-4.11) | 1.96 (0.41-12.40) | 0.91 (0.37-2.44) | 0.62 (0.15-2.37) | NA | 1.15 (0.28-4.68) | 4.59 (0.71-29.30) | 0.72 (0.20-2.60) | 1.39 (0.40-4.78) | NA | 0.70 (0.11-4.45) | 0.60 (0.10-3.55) | 0.74 (0.18-2.88) | NA | NA | 1.01 (0.45-2.18) | 2.67 (0.53-12.70) | 0.34 (0.07-1.60) | 1.11 (0.18-6.77) | 1.19 (0.41-4.42) |
| 1.02 (0.41-2.56) | 0.90 (0.40-2.11) | Ico | 1.88 (0.27-18.40) | 0.87 (0.19-4.65) | 0.60 (0.09-3.67) | NA | 1.11 (0.15-8.20) | 4.43 (0.45-44.50) | 0.69 (0.11-4.68) | 1.33 (0.21-8.99) | NA | 0.68 (0.08-5.33) | 0.58 (0.08-4.29) | 0.71 (0.14-3.78) | NA | NA | 0.97 (0.30-3.17) | 2.57 (0.32-21.00) | 0.33 (0.04-2.34) | 1.07 (0.34-3.50) | 1.14 (0.24-7.45) |
| 1.41 (0.69-2.76) | 1.24 (0.55-2.73) | 1.39 (0.45-3.98) | Afa | 0.47 (0.09-2.02) | 0.32 (0.04-1.84) | NA | 0.58 (0.06-4.74) | 2.32 (0.17-25.30) | 0.37 (0.04-2.67) | 0.71 (0.08-4.96) | NA | 0.36 (0.03-3.71) | 0.31 (0.02-2.95) | 0.38 (0.04-2.70) | NA | NA | 0.52 (0.07-2.60) | 1.34 (0.12-12.10) | 0.17 (0.02-1.22) | 0.57 (0.04-5.63) | 0.61 (0.17-2.06) |
| 1.26 (0.60-2.50) | 1.11 (0.51-2.34) | 1.24 (0.40-3.55) | 0.90 (0.35-2.26) | Dac | 0.68 (0.15-2.52) | NA | 1.26 (0.22-6.71) | 4.99 (0.60-38.20) | 0.79 (0.15-3.74) | 1.52 (0.30-6.85) | NA | 0.78 (0.10-5.47) | 0.67 (0.09-4.22) | 0.82 (0.15-3.68) | NA | NA | 1.12 (0.34-3.13) | 2.92 (0.44-17.50) | 0.37 (0.07-1.78) | 1.23 (0.16-8.25) | 1.30 (0.55-3.66) |
| 1.81 (0.87-3.69) | 1.59 (0.74-3.50) | 1.78 (0.61-5.01) | 1.29 (0.50-3.37) | 1.43 (0.55-3.93) | Osi | NA | 1.88 (0.28-14.10) | 7.52 (0.81-78.40) | 1.16 (0.19-7.89) | 2.24 (0.37-15.50) | NA | 1.13 (0.13-10.70) | 0.97 (0.12-8.21) | 1.20 (0.20-7.81) | NA | NA | 1.63 (0.40-7.20) | 4.25 (0.52-36.80) | 0.55 (0.09-3.18) | 1.81 (0.22-16.90) | 1.91 (0.49-10.10) |
| 0.62 (0.21-1.94) | 0.55 (0.16-1.90) | 0.61 (0.14-2.58) | 0.44 (0.12-1.72) | 0.49 (0.14-1.93) | 0.34 (0.09-1.31) | Naq | NA | NA | NA | NA | NA | NA | NA | NA | NA | NA | NA | NA | NA | NA | NA |
| 1.97 (0.77-5.05) | 1.74 (0.78-3.88) | 1.94 (0.61-6.06) | 1.41 (0.46-4.43) | 1.57 (0.54-4.86) | 1.09 (0.35-3.32) | 3.15 (0.73-13.60) | Erlo+Bev | 3.97 (0.38-40.90) | 0.63 (0.10-4.24) | 1.21 (0.19-8.00) | NA | 0.62 (0.06-6.04) | 0.52 (0.05-5.00) | 0.64 (0.09-4.58) | NA | NA | 0.88 (0.17-4.35) | 2.32 (0.27-18.60) | 0.30 (0.03-2.37) | 0.97 (0.10-9.65) | 1.04 (0.18-7.22) |
| 0.98 (0.23-4.19) | 0.87 (0.22-3.40) | 0.96 (0.19-4.68) | 0.70 (0.15-3.40) | 0.78 (0.17-3.79) | 0.54 (0.11-2.62) | 1.57 (0.25-9.87) | 0.50 (0.10-2.41) | Ona+Erlo | 0.16 (0.02-1.50) | 0.30 (0.03-2.76) | NA | 0.15 (0.01-1.99) | 0.13 (0.01-1.62) | 0.16 (0.02-1.55) | NA | NA | 0.22 (0.03-1.64) | 0.58 (0.05-6.60) | **0.07 (0.01-0.83)** | 0.24 (0.02-3.19) | 0.26 (0.03-2.62) |
| NA | NA | NA | NA | NA | NA | NA | NA | NA | Erlo+Tiv | 1.93 (0.33-11.90) | NA | 0.98 (0.10-9.49) | 0.84 (0.09-7.29) | 1.02 (0.15-6.61) | NA | NA | 1.40 (0.31-6.23) | 3.71 (0.48-28.80) | 0.47 (0.06-3.51) | 1.55 (0.17-14.30) | 1.65 (0.32-10.70) |
| 0.95 (0.24-3.58) | 0.84 (0.24-2.84) | 0.93 (0.20-4.08) | 0.68 (0.16-3.01) | 0.75 (0.18-3.25) | 0.53 (0.12-2.26) | 1.52 (0.27-8.65) | 0.48 (0.11-2.06) | 0.97 (0.15-6.08) | NA | Sun+Erlo | NA | 0.50 (0.05-4.61) | 0.44 (0.05-3.63) | 0.54 (0.08-3.36) | NA | NA | 0.73 (0.16-3.14) | 1.93 (0.26-13.90) | 0.24 (0.03-1.74) | 0.80 (0.09-6.93) | 0.86 (0.17-5.25) |
| 1.47 (0.48-4.51) | 1.30 (0.39-4.45) | 1.44 (0.35-5.96) | 1.05 (0.29-4.00) | 1.17 (0.33-4.47) | 0.82 (0.22-3.08) | 2.36 (0.49-11.50) | 0.75 (0.18-3.24) | 1.50 (0.24-9.40) | NA | 1.55 (0.28-9.04) | Gef+Peme | NA | NA | NA | NA | NA | NA | NA | NA | NA | NA |
| 1.00 (0.19-5.43) | 0.88 (0.17-4.80) | 0.98 (0.16-5.98) | 0.71 (0.13-4.35) | 0.79 (0.14-4.97) | 0.56 (0.10-3.22) | 0.51 (0.08-3.32) | 1.02 (0.12-8.97) | 1.05 (0.13-8.83) | NA | 0.68 (0.09-5.11) | 1.62 (0.32-7.91) | Cil+Cet+Plat | 0.85 (0.17-4.48) | 1.05 (0.32-3.61) | NA | NA | 1.44 (0.27-7.87) | 3.79 (0.34-41.80) | 0.48 (0.05-4.92) | 1.57 (0.15-17.20) | 1.69 (0.23-16.20) |
| 0.62 (0.13-3.18) | 0.55 (0.11-2.75) | 0.61 (0.11-3.44) | 0.44 (0.08-2.51) | 0.49 (0.09-2.87) | 0.34 (0.07-1.85) | 1.00 (0.15-6.96) | 0.31 (0.05-1.93) | 0.63 (0.08-5.21) | NA | 0.65 (0.09-5.02) | 0.42 (0.06-2.97) | 0.62 (0.13-3.11) | Cet+Bev+Plat | 1.23 (0.41-3.69) | NA | NA | 1.68 (0.33-3.69) | 4.39 (0.43-47.40) | 0.56 (0.06-5.15) | 1.85 (0.18-18.60) | 1.95 (0.30-17.40) |
| 0.57 (0.18-1.88) | 0.50 (0.16-1.67) | 0.56 (0.15-2.18) | 0.41 (0.11-1.57) | 0.45 (0.12-1.82) | 0.32 (0.09-1.15) | 0.91 (0.18-4.63) | 0.29 (0.07-1.23) | 0.58 (0.10-3.53) | NA | 0.60 (0.11-3.46) | 0.39 (0.08-1.97) | 0.57 (0.17-1.90) | 0.92 (0.32-2.70) | Cet+Plat | NA | NA | 1.37 (0.44-4.31) | 3.59 (0.46-28.50) | 0.46 (0.06-3.22) | 1.49 (0.20-11.60) | 1.60 (0.33-10.30) |
| 1.97 (0.54-7.15) | 1.74 (0.48-6.43) | 1.93 (0.44-8.39) | 1.40 (0.35-5.93) | 1.56 (0.38-6.79) | 1.09 (0.27-4.44) | 3.15 (0.57-17.80) | 1.00 (0.22-4.56) | 2.00 (0.31-13.40) | NA | 2.06 (0.35-12.90) | 1.33 (0.24-7.36) | 1.97 (0.26-14.10) | 3.17 (0.45-21.80) | 3.44 (0.68-17.10) | Erlo+Plat | NA | NA | NA | NA | NA | NA |
| 0.86 (0.24-3.09) | 0.76 (0.22-2.75) | 0.84 (0.20-3.67) | 0.61 (0.15-2.54) | 0.68 (0.17-2.94) | 0.48 (0.12-1.88) | 1.37 (0.25-7.60) | 0.43 (0.10-1.99) | 0.87 (0.14-5.74) | NA | 0.91 (0.16-5.46) | 0.58 (0.11-3.13) | 1.38 (0.20-9.63) | 1.50 (0.30-7.67) | 0.44 (0.08-2.37) | 1.74 (0.53-5.72) | Mot+Plat | NA | NA | NA | NA | NA |
| **0.49 (0.31-0.80)** | **0.44 (0.28-0.71)** | 0.48 (0.21-1.11) | **0.35 (0.17-0.76)** | **0.39 (0.19-0.89)** | **0.27 (0.14-0.55)** | 0.79 (0.24-2.69) | **0.25 (0.10-0.63)** | 0.50 (0.12-2.15) | NA | 0.52 (0.14-1.99) | 0.33 (0.10-1.10) | 0.49 (0.10-2.45) | 0.79 (0.17-3.62) | 0.86 (0.29-2.55) | 0.25 (0.08-0.85) | 0.57 (0.18-1.88) | Plat | 2.64 (0.45-15.20) | 0.34 (0.07-1.65) | 1.10 (0.21-5.77) | 1.18 (0.37-4.82) |
| 0.94 (0.25-3.56) | 0.82 (0.24-2.79) | 0.91 (0.21-3.96) | 0.67 (0.16-2.91) | 0.74 (0.18-3.21) | 0.51 (0.12-2.21) | 1.49 (0.26-8.52) | 0.47 (0.11-2.03) | 0.95 (0.15-5.96) | NA | 0.98 (0.17-5.66) | 0.63 (0.11-3.59) | 0.93 (0.12-7.40) | 1.50 (0.20-11.10) | 1.64 (0.29-8.81) | 0.48 (0.08-2.84) | 1.08 (0.18-6.25) | 1.89 (0.51-6.92) | Doc | 0.13 (0.01-1.19) | 0.42 (0.04-4.59) | 0.45 (0.07-3.60) |
| 3.16 (0.85-11.60) | 2.79 (0.69-11.30) | 3.08 (0.61-15.20) | 2.24 (0.52-10.00） | 2.50 (0.57-11.40) | 1.75 (0.39-7.77) | 5.04 (0.90-28.30) | 1.59 (0.32-7.92) | 3.21 (0.45-22.7) | NA | 3.30 (0.52-21.90) | 2.13 (0.38-11.80) | 3.13 (0.37-26.40) | 5.07 (0.64-39.00) | 5.49 (0.92-31.60) | 1.60 (0.25-10.10) | 3.68 (0.58-22.30) | 6.40 (1.57-25.30) | 3.40 (0.51-21.80) | Vin | 3.30 (0.34-34.70) | 3.55 (0.73-22.40) |
| 0.57 (0.14-2.59) | 0.50 (0.13-2.17) | 0.56 (0.18-1.81) | 0.41 (0.09-2.11) | 0.45 (0.10-2.34) | 0.32 (0.08-1.55) | 0.92 (0.15-5.88) | 0.29 (0.06-1.52) | 0.58 (0.08-4.29) | NA | 0.60 (0.09-4.14) | 0.39 (0.06-2.47) | 0.57 (0.07-4.89) | 0.93 (0.12-7.39) | 1.00 (0.17-5.97) | 0.29 (0.05-1.92) | 0.67 (0.11-4.29) | 1.15 (0.28-4.91) | 0.61 (0.10-4.08) | 0.18 (0.03-1.36) | WBRT | 1.06 (0.15-9.99) |
| **0.52 (0.28-0.91)** | **0.46 (0.21-0.93)** | 0.51 (0.17-1.40) | **0.37 (0.17-0.78)** | **0.41 (0.19-0.88)** | **0.29 (0.11-0.70)** | 0.83 (0.22-2.81) | **0.26 (0.08-0.75)** | 0.53 (0.11-2.41) | NA | 0.55 (0.13-2.26) | 0.35 (0.10-1.19) | 0.52 (0.08-2.88) | 0.84 (0.14-4.18) | 0.91 (0.23-3.16) | 0.26 (0.06-1.03) | 0.60 (0.14-2.32) | 1.06 (0.49-2.06) | 0.55 (0.13-2.25) | **0.17 (0.04-0.67)** | 0.91 (0.17-4.04) | Placebo |
| Results for PFS are shown in blue-colour cells, results for OS are in gray-color cells. Comparisons should be read from left to right and the estimate is in the cell in common between the column-defining drugs and the row-defining treatment. For PFS and OS, HRs (and 95% CI) less than 1 favour the column-defining treatment. To obtain HRs for comparisons in the opposite direction, reciprocals should be taken. Significant results are in bold and underscored. Abbreviations: Gef, gefitinib; Erlo, erlotinib; Ico, icotinib; Afa, afatinib; Dac, dacomitinib; Osi, osimertinib; Naq, naquotinib; Erlo+Bev, erlotinib+bevacizumab; Ona+Erlo, onartuzumab+erlotinib; Erlo+Tiv, erlotinib+tivantinib; Sun+Erlo, sunitinib+erlotinib; Gef+Peme, gefitinib+pemetrexed; Cil+Cet+Plat, cilengitide+cetuximab+platinum-based therapy; Cet+Bev+Plat, cetuximab+bevacizumab+platinum-based therapy; Cet+Plat, cetuximab+platinum-based therapy; Erlo+Plat, erlotinib+platinum-based therapy; Mot+Plat, motesanib+platinum-based therapy; Plat, platinum-based therapy; Doc, docetaxel; Vin, vinorelbine; WBRT, whole-brain radiotherapy; PFS, progression-free survival; OS, overall survival; HR, hazard-ratio, CI, confidence interval. | | | | | | | | | | | | | | | | | | | | | |
|
|
|
|
