# Supplementary material for: Therapeutic options for advanced epidermal growth factor receptor (EGFR)-mutant non-small cell lung cancer: a Bayesian network secondary analysis
Source: Aging (Albany NY). 2020 Apr 23;12(8):7129–62. doi: 10.18632/aging.103066 (PMC7202525; doi:10.18632/aging.103066)
Supplement: Appendix Table 5 [file aging-12-103066-s002..doc]

**Appendix Table A5.** Efficacy of all available interventions according to class-level Bayesian network analysis

| 1st-gen ET | 0.96 (0.54-2.14) | 0.63 (0.24-1.68) | 1.30 (0.56-2.98) | 1.14 (0.47-3.43) | NA | NA | 0.74 (0.24-2.20) | NA | 0.63 (0.18-2.38) | NA | 1.01 (0.58-1.72) | 0.77 (0.30-2.09) | 1.07 (0.39-2.97) | 0.97 (0.48-2.50) |
| --- | --- | --- | --- | --- | --- | --- | --- | --- | --- | --- | --- | --- | --- | --- |
| 1.25 (0.73-2.07) | 2nd-gen ET | 0.66 (0.18-1.92) | 1.34 (0.41-3.58) | 1.17 (0.38-3.90) | NA | NA | 0.77 (0.19-2.47) | NA | 0.66 (0.14-2.59) | NA | 1.05 (0.39-2.24) | 0.78 (0.24-2.48) | 1.12 (0.29-3.40) | 1.01 (0.52-2.00) |
| 1.27 (0.69-2.32) | 1.02 (0.47-2.26) | 3rd-gen ET | 2.06 (0.56-7.47) | 1.79 (0.52-8.36) | NA | NA | 1.17 (0.26-5.15) | NA | 1.00 (0.20-5.26) | NA | 1.59 (0.52-4.90) | 1.20 (0.33-5.08) | 1.71 (0.42-7.03) | 1.52 (0.49-6.31) |
| 1.42 (0.71-2.83) | 1.14 (0.49-2.75) | 1.12 (0.44-2.82) | ET+aVEGFR | 0.88 (0.26-3.56) | NA | NA | 0.56 (0.15-2.26) | NA | 0.48 (0.11-2.31) | NA | 0.77 (0.29-2.10) | 0.59 (0.18-2.23) | 0.83 (0.22-3.08) | 0.75 (0.26-2.75) |
| 0.87 (0.21-3.51) | 0.70 (0.16-3.09) | 0.68 (0.15-3.18) | 0.62 (0.13-2.88) | MT+ET | NA | NA | 0.66 (0.13-2.40) | NA | 0.56 (0.10-2.46) | NA | 0.89 (0.26-2.41) | 0.67 (0.17-2.46) | 0.95 (0.20-3.42) | 0.86 (0.24-2.98) |
| 0.63 (0.11-3.65) | 0.51 (0.08-3.09) | 0.50 (0.08-3.13) | 0.45 (0.07-2.95) | 0.73 (0.08-7.14) | IT+Plat | NA | NA | NA | NA | NA | NA | NA | NA | NA |
| 1.19 (0.20-6.93) | 0.95 (0.15-5.94) | 0.94 (0.15-5.92) | 0.84 (0.13-5.58) | 1.36 (0.15-13.40) | 1.87 (0.53-6.70) | IT+aVEGFR+Plat | NA | NA | NA | NA | NA | NA | NA | NA |
| 0.92 (0.44-1.97) | 0.74 (0.31-1.88) | 0.73 (0.30-1.88) | 0.65 (0.24-1.85) | 1.07 (0.22-5.26) | 1.45 (0.24-9.65) | 0.77 (0.12-5.08) | ET+Plat | NA | 0.85 (0.43-1.78) | NA | 1.37 (0.53-3.58) | 1.03 (0.26-4.72) | 1.46 (0.33-6.42) | 1.31 (0.38-5.88) |
| 0.73 (0.20-2.64) | 0.58 (0.15-2.31) | 0.57 (0.15-2.28) | 0.51 (0.12-2.21) | 0.84 (0.13-5.74) | 1.14 (0.35-3.77) | 0.61 (0.18-2.04) | 0.79 (0.19-3.16) | aVEGFR+Plat | NA | NA | NA | NA | NA | NA |
| 1.24 (0.42-3.85) | 1.00 (0.30-3.44) | 0.87 (0.24-3.38) | 1.44 (0.24-8.64) | 1.96 (0.27-15.20) | 1.05 (0.14-8.14) | 1.35 (0.59-3.07) | 1.71 (0.34-9.10) | 0.84 (0.17-4.34) | ET+aVEGFR+Plat | NA | 1.60 (0.48-5.10) | 1.20 (0.26-6.20) | 1.70 (0.32-8.68) | 1.54 (0.37-7.99) |
| 1.46 (0.47-4.73) | 1.17 (0.34-4.28) | 1.16 (0.32-4.41) | 1.04 (0.28-4.09) | 1.69 (0.28-10.60) | 2.32 (0.28-19.00) | 1.24 (0.15-10.40) | 1.60 (0.40-6.39) | 2.02 (0.36-11.40) | 1.19 (0.23-5.84) | ET+CT | NA | NA | NA | NA |
| **0.41 (0.28-0.61)** | **0.33 (0.19-0.62)** | **0.33 (0.18-0.61)** | **0.29 (0.13-0.65)** | 0.48 (0.11-2.06) | 0.65 (0.12-3.63) | 0.35 (0.06-1.98) | **0.45 (0.22-0.90)** | 0.57 (0.17-1.94) | **0.33 (0.11-0.97)** | **0.28 (0.08-0.94)** | Plat | 0.76 (0.27-2.43) | 1.07 (0.34-3.40) | 0.97 (0.41-2.89) |
| 1.53 (0.61-3.87) | 1.23 (0.43-3.57) | 1.21 (0.41-3.67) | 1.08 (0.34-3.48) | 1.77 (0.33-9.37) | 2.42 (0.34-17.60) | 1.29 (0.18-9.49) | 1.66 (0.50-5.38) | 2.11 (0.43-10.30) | 1.23 (0.29-5.19) | 1.04 (0.23-4.53) | 3.69 (1.34-10.01) | CT | 1.41 (0.33-5.34) | 1.29 (0.38-4.70) |
| 0.56 (0.17-1.85) | 0.45 (0.13-1.68) | 0.44 (0.12-1.67) | 0.40 (0.10-1.59) | 0.65 (0.11-4.08) | 0.88 (0.11-7.50) | 0.47 (0.06-4.04) | 0.61 (0.15-2.47) | 0.77 (0.14-4.51) | 0.45 (0.09-2.24) | 0.38 (0.07-2.00) | 1.35 (0.40-4.75) | 1.13 (0.31-4.46) | WBRT | 0.90 (0.27-3.77) |
| **0.50 (0.27-0.89)** | **0.40 (0.21-0.73)** | **0.39 (0.16-0.91)** | **0.35 (0.14-0.85)** | 0.57 (0.12-2.59) | 0.79 (0.12-4.90) | 0.42 (0.06-2.63) | 0.54 (0.20-1.36) | 0.69 (0.16-2.72) | 0.40 (0.11-1.37) | 0.34 (0.09-1.19) | 1.20 (0.58-1.19) | **0.32 (0.10-0.95)** | 0.88 (0.22-3.27) | Placebo |

Results for PFS are shown in blue-colour cells, results for OS are in gray-color cells. Comparisons should be read from left to right and the estimate is in the cell in common between the column-defining drugs and the row-defining treatment. For PFS and OS, HRs (and 95% CI) less than 1 favour the column-defining treatment. To obtain HRs for comparisons in the opposite direction, reciprocals should be taken. Significant results are in bold and underscored. Abbreviations: 1st-gen ET, first generation EGFR-TKI; 2nd-gen ET, second generation EGFR-TKI; 3rd-gen ET, third generation EGFR-TKI; ET+aVEGFR, EGFR-TKI+anti-VEGFR; MT+ET, MET-TKI+EGFR-TKI; IT+Plat, immunotherapy+platinum-based therapy; IT+aVEGFR+Plat, immunotherapy+anti-VEGFR+platinum-based therapy; ET+Plat, EGFR-TKI+platinum-based therapy; aVEGFR+Plat, anti-VEGFR+platinum-based therapy; ET+aVEGFR+Plat, EGFR-TKI+anti-VEGFR+platinum-based therapy; ET+CT, EGFR-TKI+cytotoxic therapy; Plat, platinum-based therapy; CT, cytotoxic therapy, WBRT, whole-brain radiotherapy; PFS, progression-free survival; OS, overall survival; HR, hazard-ratio, CI, confidence interval.
